# A functional regulatory variant of *MYH3* influences muscle fiber-type composition and intramuscular fat content in pigs

**DOI:** 10.1371/journal.pgen.1008279

**Published:** 2019-10-11

**Authors:** In-Cheol Cho, Hee-Bok Park, Jin Seop Ahn, Sang-Hyun Han, Jae-Bong Lee, Hyun-Tae Lim, Chae-Kyoung Yoo, Eun-Ji Jung, Dong-Hwan Kim, Wu-Sheng Sun, Yuliaxis Ramayo-Caldas, Sang-Geum Kim, Yong-Jun Kang, Yoo-Kyung Kim, Hyun-Sook Shin, Pil-Nam Seong, In-Sul Hwang, Beom-Young Park, Seongsoo Hwang, Sung-Soo Lee, Youn-Chul Ryu, Jun-Heon Lee, Moon-Suck Ko, Kichoon Lee, Göran Andersson, Miguel Pérez-Enciso, Jeong-Woong Lee

**Affiliations:** 1 National Institute of Animal Science, Rural Development Administration, Jeju, Republic of Korea; 2 Department of Animal Resources Science, College of Industrial Sciences, Kongju National University, Yesan, Republic of Korea; 3 Biotherapeutics Translational Research Center, Korea Research Institute of Bioscience and Biotechnology, Daejeon, Republic of Korea; 4 Educational Science Research Institute, Jeju National University, Jeju, Republic of Korea; 5 Korea Zoonosis Research Institute, Chonbuk National University, Iksan, Republic of Korea; 6 Department of Animal Science, College of Agriculture and Life Sciences, Gyeongsang National University, Jinju, Republic of Korea; 7 Institute of Agriculture and Life Science, Gyeongsang National University, Jinju, Republic of Korea; 8 Bio-Medical Science Co., Ltd., Gimpo, Republic of Korea; 9 Department of Functional Genomics, University of Science and Technology, Daejeon, Republic of Korea; 10 Génétique Animale et Biologie Intégrative (GABI), INRA, AgroParisTech, Université Paris-Saclay, Jouy-en-Josas, France; 11 Animal Breeding and Genetics Program, Institute for Research and Technology in Food and Agriculture (IRTA), Torre Marimon, Caldes de Montbui, Spain; 12 National Institute of Animal Science, Rural Development Administration, Wanju, Republic of Korea; 13 National Institute of Animal Science, Rural Development Administration, Namwon, Republic of Korea; 14 Division of Biotechnology, SARI, Jeju National University, Jeju, Republic of Korea; 15 Division of Animal and Dairy Science, Chungnam National University, Deajeon, Republic of Korea; 16 Department of Animal Sciences, College of Food, Agricultural, and Environmental Sciences, The Ohio State University, Columbus, OH, United States of America; 17 Department of Animal Breeding and Genetics, Swedish University of Agricultural Sciences, Uppsala, Sweden; 18 Centre for Research in Agricultural Genomics (CRAG), CSIC-IRTA-UAB-UB Consortium, Barcelona, Spain; 19 Departament de Ciència Animal i dels Aliments, Universitat Autònoma de Barcelona, Barcelona, Spain; 20 ICREA, Carrer de Lluís Companys, Barcelona, Spain; University of Bern, SWITZERLAND

## Abstract

Muscle development and lipid accumulation in muscle critically affect meat quality of livestock. However, the genetic factors underlying myofiber-type specification and intramuscular fat (IMF) accumulation remain to be elucidated. Using two independent intercrosses between Western commercial breeds and Korean native pigs (KNPs) and a joint linkage-linkage disequilibrium analysis, we identified a 488.1-kb region on porcine chromosome 12 that affects both reddish meat color (a*) and IMF. In this critical region, only the *MYH3* gene, encoding myosin heavy chain 3, was found to be preferentially overexpressed in the skeletal muscle of KNPs. Subsequently, *MYH3*-transgenic mice demonstrated that this gene controls both myofiber-type specification and adipogenesis in skeletal muscle. We discovered a structural variant in the promotor/regulatory region of *MYH3* for which *Q* allele carriers exhibited significantly higher values of a* and IMF than *q* allele carriers. Furthermore, chromatin immunoprecipitation and cotransfection assays showed that the structural variant in the 5′-flanking region of *MYH3* abrogated the binding of the myogenic regulatory factors (MYF5, MYOD, MYOG, and MRF4). The allele distribution of *MYH3* among pig populations worldwide indicated that the *MYH3 Q* allele is of Asian origin and likely predates domestication. In conclusion, we identified a functional regulatory sequence variant in porcine *MYH3* that provides novel insights into the genetic basis of the regulation of myofiber type ratios and associated changes in IMF in pigs. The *MYH3* variant can play an important role in improving pork quality in current breeding programs.

## Author summary

Local native livestock breeds, such as pigs and cattle, are important reservoirs of genetic variation, because they have adapted to local conditions and have not been as strongly selected as commercial livestock breeds. Therefore, the study of local native breeds is of utmost interest to identify causative mutations for traits in which large differences exist between local native breeds and breeds used internationally for meat production. Herein, we apply these principles to meat quality related traits in pigs. A combined linkage-linkage disequilibrium analysis identified the *MYH3* gene on pig chromosome 12, which encodes myosin heavy chain 3 protein. Subsequently, we discovered a regulatory mutation in *MYH3*, which occurs at high frequency in Korean Native Pigs compared to the frequency at which it occurs in Western pig breeds. We confirmed the effect of *MYH3* on muscle fiber-type composition and intramuscular fat content using transgenic mice and transfection assays in porcine fibroblast cells. This is the first causal mutation identified in livestock for these traits and the result is immediately applicable for the genetic improvement of pork quality. These results also enhance our understanding of the genetic basis regarding the regulation of myofiber type ratios and associated changes in intramuscular fat contents in pigs and mammals in general.

## Introduction

**Fig 1:**
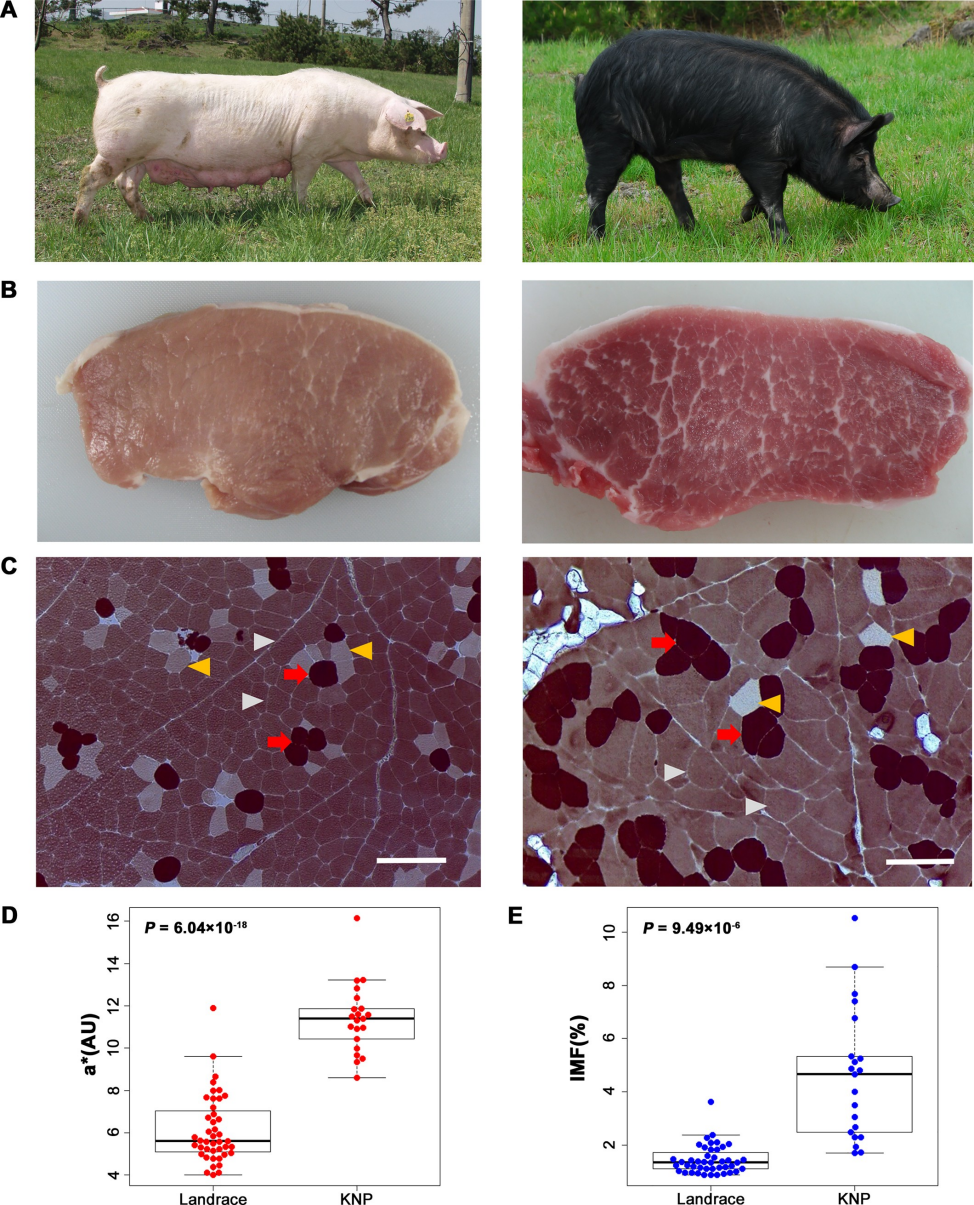
Pigs used in this study. (**A**) Photos of a Landrace pig (left) and a KNP (right). (**B**) Photos of cross section of *longissimus dorsi* muscle from the Landrace (left) and KNP (right). Note the pronounced difference in reddish meat color (a*) as well as marbling (i.e., intramuscular fat, IMF). (**C**) Myosin ATPase histochemistry after preincubation at pH 4.6 of the *longissimus dorsi* muscle from the Landrace (left) and KNP (right). Red arrows indicate type1 (slow/oxidative) fiber; yellow arrow heads indicate type2A fiber (fast/oxido-glycolytic); gray arrowheads indicate type2B fiber (fast/glycolytic). Scale bar = 200 μm. (**D**) Boxplot with individual raw a* values of Landrace (n = 43) and KNP (n = 21) (**E**) Boxplot with individual raw IMF contents of Landrace (n = 43) and KNP (n = 21).

Despite the remarkable progress of studies using high-throughput genome technologies combined with genome-wide linkage and association in various organisms, elucidating the genetic architecture of complex quantitative traits remains a key challenge of modern biology [1]. In this respect, the phenotypic and genetic diversity among breeds of domestic animals provides an excellent opportunity to investigate the relationship between phenotypic and genotypic variations [2, 3]. Despite their retarded growth, Korean native black pigs (KNPs) found on Jeju Island are renowned for their meat quality characteristics, such as a reddish meat color (a*) and a high degree of marbling (i.e., intramuscular fat, IMF) compared with the traits of the Western commercial pig breeds such as Landrace (Fig 1A and 1B) [4]. The clear a* of KNPs is mainly due to the large amount of slow/type1/oxidative myofibers in the muscle tissue (Fig 1C and 1D), and the high degree of marbling is associated with the excess accumulation of IMF (Fig 1E). The variation in muscle fiber composition and IMF content is complex; highly interrelated phenomena are dependent on multiple genetic components and, environmental conditions together with a host of various cellular signals and hormones involved in myogenesis and adipogenesis [5, 6]. Therefore, it is expected that muscle fiber- and IMF- related traits are remarkably complex quantitative traits, of critical economic importance, but the underlying genetic basis is largely unknown. Here, linkage and association analyses were conducted to dissect the genetic architecture of a* and IMF traits in pork, and a major QTL on chromosome 12 was identified to be significantly associated with the two traits. The aim of this study was to elucidate and characterize the genetic determinant underlying the major QTL.

## Results

### Identification of the porcine *MYH3* gene as a putative quantitative trait gene (QTG) for a* and IMF content

To investigate the genetic basis underlying a* and IMF in the *longissimus dorsi* muscle, we generated a large intercross between Landrace pigs and KNPs with 1,105 F_2_ progeny (LK cross) [7] and identified a quantitative trait locus (QTL) located on pig chromosome 12 (SSC12) that had a substantial effect on both a* and IMF based on genome-wide linkage analysis [8]. Previous studies reported that the QTL on SSC12 contains a cluster of genes encoding the myosin heavy chains (MYHs) which are strongly associated with a* and IMF [9, 10]. Here, we genotyped the entire LK cross using the Illumina PorcineSNP60K BeadChip platform [11, 12]. A genome-wide association study (GWAS) revealed a major locus for the two traits [a*: 26.6% phenotypic variance explained by the SNP (% Var_SNP_), *P*-value = 1.5×10^−70^; IMF: 24.2% Var_SNP_, *P*-value = 1.1×10^−88^] at the SSC12 region harboring the *MYH* gene cluster (S1A and S1B Fig). The position of the most significantly associated marker (rs81437379) for the two traits was 54,956,054 (NCBI *Sus scrofa* version 11.1). Using linkage analysis, instead of an association study, we could also replicate the major QTL for the two traits at rs81437379 (S2A Fig; a*: 20.5% Var_QTL_, *P*-value = 1.02×10^−49^; IMF: 29.4% Var_QTL_, *P*-value = 3.2×10^−74^).

**Fig 2:**
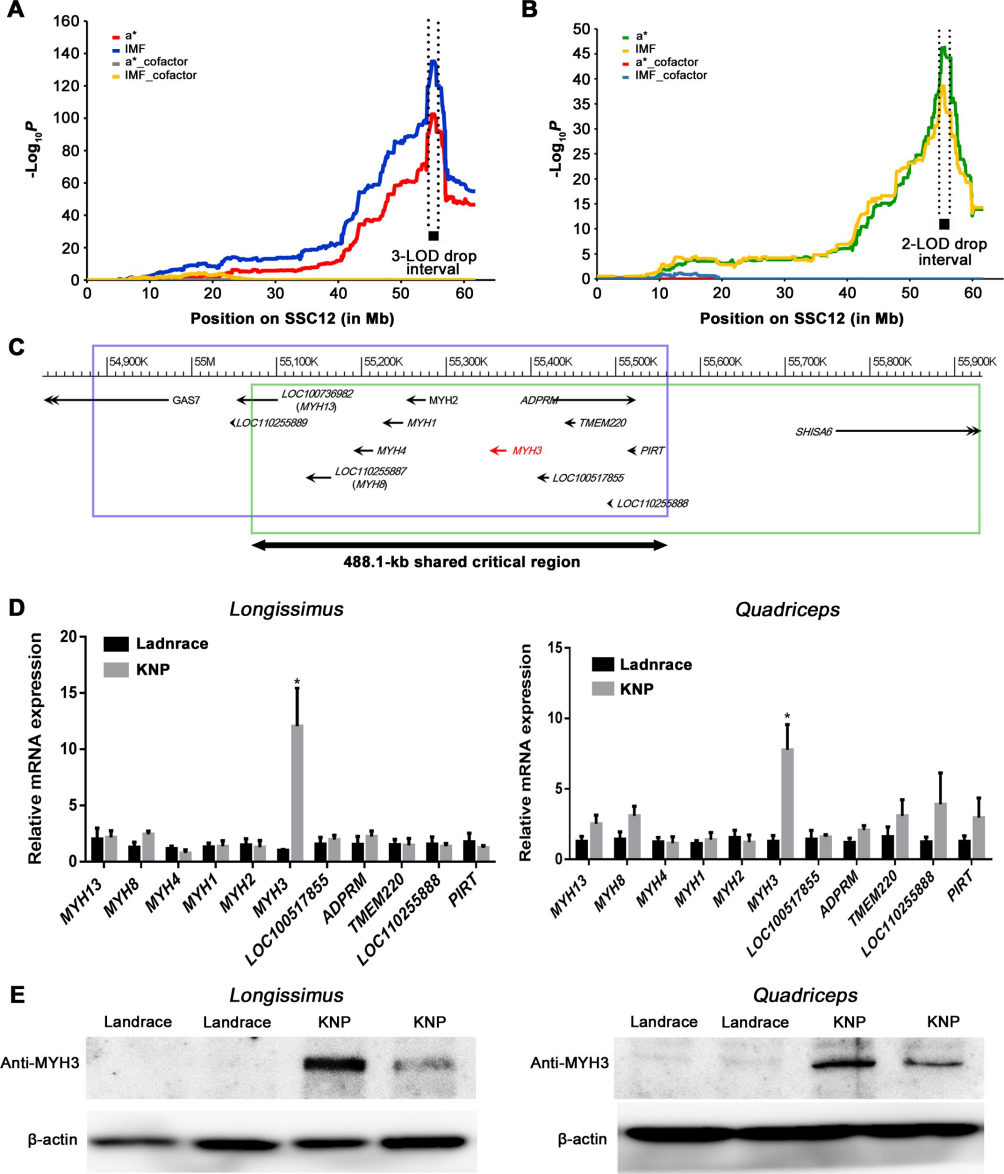
High-resolution mapping of a QTL that affects a* and IMF contents in the *longissimus dorsi* muscles of LK (n = 1,232) and DK (n = 395) crosses. (**A**) LALD mapping results on SSC12 for a* and IMF from the LK cross. In the case of a* and IMF, the LALD mapping with correction for the effect of the most likely QTL did not show any sign of additional QTL on SSC12 (yellow and gray dotted lines). (**B**) LALD mapping results on SSC12 for a* and IMF from the DK cross. The vertical dotted lines for each cross were estimated by the LOD-drop method (S3 Fig). (**C**) Blue (LK cross, 3-LOD drop support interval) and green (DK cross, 2-LOD drop support interval) boxes indicate cross-specific LOD-drop support intervals. Maximum test statistics for each cross were obtained at the region colocalized in the 488.1-kb critical shared region represented by the black double headed arrow (12: 55,073,130–55,561,243). Eleven NCBI protein coding genes are located within the 488.1-kb critical interval associated with a* and IMF. Gene names in parentheses were annotated by this study (S1 Table and S4 Fig). (**D**) Gene transcription analysis of the 11 positional candidate genes. Relative mRNA expression levels of the 11 genes in the *longissimus dorsi* muscle (left) and in the *quadriceps* muscle (right) in Landrace (n = 6) and KNP (n = 6). Data histograms and error bars represent the mean±standard error, *P<0.05. (**E**) Western blotting analysis of MYH3 in the *longissimus dorsi* muscle (left) and in the *quadriceps* muscle (right) between Landrace and KNP. We used a muscle sample from one animal per lane.

To refine the identified QTL on SSC12 in the LK cross, we conducted a joint linkage and linkage disequilibrium (LALD) analysis of a* and IMF using DualPHASE software [13]. The test statistics of the LALD mapping for a* (*P*-value = 3.47×10^−103^) and IMF (*P*-value = 7.35×10^−136^) were maximized at the 718.4-kb QTL region (i.e., 12:54,842,795–55,561,243, Fig 2A and 2C; S3 Fig). The effects of founder haplotypes (i.e., those haplotypes found in the parental animals) were estimated at the most likely QTL position. The distribution of the founder haplotype effects formed a bimodal cluster, which supported a biallelic QTL model (S5A and S5C Fig).

To confirm the QTL signals identified in the LK cross, we established another independent intercross between Duroc pigs and KNPs with 381 F_2_ offspring (DK cross). All of the individuals in this cohort were also genotyped using the Illumina PorcineSNP60K BeadChip in a manner similar to the genotyping of the LK cross. The additional GWAS and linkage analysis replicated the finding of the highly significant association and linkage signals for a* (*P*-value for association = 7.2×10^−11^; *P*-value for linkage = 1.2×10^−16^) and IMF (*P*-value for association = 4.3×10^−13^; *P*-value for linkage = 3.2×10^−19^) at the same location (rs81437379) on SSC12 as detected in the LK cross (S1C and S1D Fig; S2B Fig). Furthermore, the LALD mapping using the DK cross revealed the maximum test statistics at the region of the 858.6-kb interval for a* and IMF (12:55,073,130–55,931,714; Fig 2B and 2C; S3 Fig). The distribution of the founder haplotype effects from the LALD mapping also showed a bimodal shape in the DK cross (S5B and S5D Fig). Subsequently, a conservative 2-LOD drop support interval was applied to define the shared critical region identified by LALD mapping in the LK and DK crosses as described in S3 Fig. The interval size of the new critical region was 488.1-kb (12:55,073,130–55,561,243). According to the NCBI *Sus scrofa* 11.1 annotation, the critical interval contained eleven protein coding genes, including *LOC100736982*, *LOC110255887*, *MYH4*, *MYH1*, *MYH2*, *MYH3*, *LOC100517855*, *ADPRM*, *TMEM220*, *LOC110255888* and *PIRT* (Fig 2C). Based on comparative sequence and phylogenetic analyses, *LOC100736982* and *LOC110255887* were identified as *MYH13* and *MYH8*, respectively (S1 Table and S4 Fig).

In both the *longissimus* and *quadriceps* muscles, quantitative reverse transcriptase-PCR (qRT-PCR) detected a highly significant difference in *MYH3* transcript level abundance between KNPs and Landrace pigs, with this gene being transcribed approximately 7-12-fold more actively in KNPs than in Landrace pigs (Fig 2D). The results of Western blotting analysis using proteins prepared from the skeletal muscle samples confirmed the differential expression of MYH3 between the two pig breeds (Fig 2E). We did not detect any significant differences in transcription levels in any of the other genes located within this critical interval.

### Phenotypic changes induced by ectopic expression of porcine MYH3 in transgenic (TG) mice mimic the KNP phenotype

**Fig 3:**
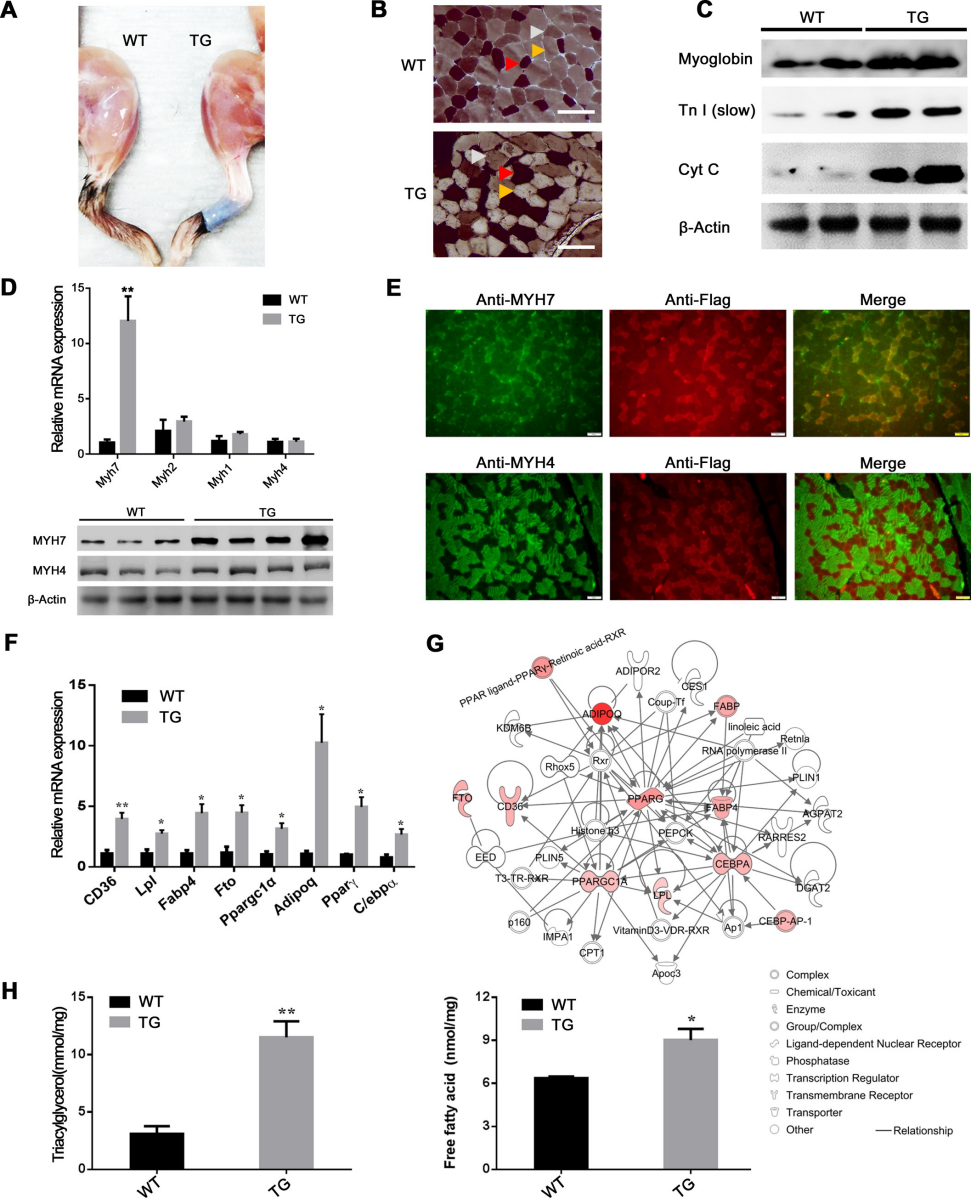
Characterization of *MYH3* TG mice for muscle fiber type specification and adipogenesis. (**A**) Gross morphology of hindlimb muscle of WT and TG mice. (**B**) Hindlimb muscle stained for myosin ATPase histochemistry. Red arrowheads indicate type1 (slow/oxidative) fiber; yellow arrowheads indicate type2A (fast/oxido-glycolytic); and gray arrowheads indicate type2B (fast/glycolytic). Scale bar = 50 μm. (**C**) Western blotting assays of slow-type muscle associated proteins extracted from *quadriceps* muscles. We used a muscle sample from one animal per lane. (**D**) Gene expression analyses of muscle fiber type-associated genes by qRT-PCR (upper) and Western blot (lower). Data are from four-months-old WT (n = 3) and TG (n = 5) mice. (**E**) Immunohistochemical analysis using anti-MYH4 and anti-MYH7 antibodies in TG mice. Scale bar = 50 μm. (**F**) Expression of eight adipogenesis-associated genes in *quadriceps* muscle by qRT-PCR. Data are from mRNA prepared from four-month-old WT (n = 3) and TG (n = 5) mice. Data are presented as the mean±standard error. *P<0.05, **P<0.01. (**G**) The gene interaction network for adipogenesis generated by the ingenuity pathway analysis (IPA). Overexpressed genes are labeled in a reddish color. The color concentration represents the fold change of the genes (*e*.*g*., *Adipoq* shows the highest fold change). (**H**) Measurement of triacylglycerol and free fatty acids in *quadriceps* muscle. Data are from four-month-old WT (n = 3) and TG (n = 5) mice. *P<0.05, **P<0.01.

To evaluate the role of MYH3 in muscle fiber composition and lipogenesis in skeletal muscle, we generated TG mice overexpressing porcine MYH3 (S6A and S6B Fig). No significant body weight difference was observed between TG mice and wild-type (WT) mice (S6C Fig). From a morphological aspect, the hindlimb of the TG-mouse strain number 24, which exhibited the highest MYH3 expression among the four TG-mouse strains (S6A and S6B Fig), showed a recognizable reddish color that is characteristic of slow/type1/oxidative muscle fiber, while the counterpart of the WT-mouse strain was paler in appearance, strongly suggesting the influence of MYH3 on muscle fiber-type composition (Fig 3A). Accordingly, myosin ATPase staining analysis revealed a greater presence of slow/type1/oxidative fibers in the *quadriceps* muscles of TG mice, whereas the *quadriceps* muscles of the WT mice mainly contained fast- type fibers (fast/type2A/oxido-glycolytic and fast/type2B/glycolytic) (Fig 3B). In particular, a significant increase in the area of the slow type muscle fiber was detected (*P*-value = 0.0004) in TG mice compared to that of the WT mice, whereas, no significant difference (*P*-value = 0.93) in the area of the fast-type fibers was found (S6D Fig).

The TG-mice overexpressing MYH3 also showed enhanced levels of myoglobin, troponin I (Tn I), and mitochondrial oxidative cytochrome c enzyme (Cyt C), all of which are critical features of slow/type1/oxidative muscle fibers (Fig 3C). Messenger-RNA expression of *Myh7*, which is a molecular marker for slow/type1/oxidative fiber [14], was higher in the TG mice than in the WT mice (*P*-value < 0.05). However, the three molecular markers for fast-type muscle fibers (i.e., *Myh1*, *Myh2*, and *Myh4* [14]), showed no significant differential mRNA expression between the two strains of mice (Fig 3D). Western blotting analysis of Myh7 and Myh4 also demonstrated the same expression pattern as their transcripts (Fig 3D). The results of immunohistochemical analysis showed a high degree of colocalization of porcine MYH3 and slow Myh7 in the TG-mice, while the opposite localization of porcine MYH3 and fast Myh4 was detected (Fig 3E). Moreover, qRT-PCR analysis revealed strongly increased gene expression of slow/type1/oxidative fiber associated genes (*Myoglobin*, *Tnnt1*, *Tnni1*, and *Tnnc1*). However, no significant expression difference in fast-type muscle fiber-associated genes (*Aldoa*, *Pvalb*, *Tnnt3*, *Tnni2*, and *Tnnc2*) was observed between TG and WT mice (S6E Fig). Combined, these results clearly indicate that overexpression of *MYH3* influences the myofiber composition in the skeletal muscle of the TG mice.

The way in which MYH3 functionally influences the expression of adipogenesis-related genes is still unknown. Nevertheless, we selected eight genes actively involved in adipogenesis (i.e., *CD36*, *Lpl*, *Fabp4*, *Fto*, *Ppargc1α*, *Adipoq*, *Pparγ*, and *C/ebpα*) to test whether the overexpression of the *MYH3* gene influences the expression of genes known to be involved in the adipogenesis pathway in skeletal muscle [15–17]. The eight adipogenesis-related genes displayed significantly higher mRNA expression in TG mice compared to WT mice (Fig 3F). Furthermore, the ingenuity pathway analysis (IPA) was used to infer a molecular interaction network of adipogenesis (Fig 3G). The IPA also identified the adipogenesis pathway as the top-ranked canonical pathway with strong statistical support (*P-*value = 1.02×10^−7^). These results suggest that the overexpression of MYH3 regulates the coordinated expression of genes involved in adipogenesis in the skeletal muscle tissues of the TG mice partially due to increased IMF resulting from the more abundant myofiber type I. MYH3 overexpression in *quadriceps* skeletal muscles also enhanced levels of intramyocellular triacylglycerol (TAG) and free fatty acids (FFAs) (Fig 3H). These data further support *MYH3* as the most likely QTG responsible for the regulation of myofiber type ratios and the associated changes in adipogenesis in the skeletal muscle of both mice and pigs.

### Sequencing F_1_ sire chromosomes, marker-assisted segregation analysis (MASA), and *in silico* functional annotation detect putative functional sequence variants (FSVs) of *MYH3* affecting a* and IMF

To identify FSVs in the 488.1-kb critical region that affect the porcine *MYH3* gene (S3 Fig), we first sequenced genomic DNA samples from the F_1_ sires of LK (n = 18) and DK (n = 6) crosses together with the parental animals using a massively parallel sequencing technology [i.e., Landrace (n = 17) and KNP (n = 19) for LK cross; Duroc (n = 9) and KNP (n = 5) for DK cross]. The criteria to detect putative FSVs among the identified DNA sequence variants (DSVs) within the 488.1-kb critical interval were as follows: 1) the FSV has to be biallelic since the effect of the founder haplotypes followed a bimodal distribution (S5 Fig); 2) it should be segregating in both LK and DK crosses; and 3) it should affect the binding capacity of regulatory sequence motifs i.e., transcription factor binding sites such as the enhancer, silencer and promoter in the noncoding region, because we detected a clear differential *MYH3* expression between the two parental breeds (Fig 2D and 2E). In this 488.1-kb critical region, we detected 7,606 DSVs from the LK cross and 5,211 DSVs from the DK cross. Using 12 (LK cross) and 5 (DK cross) informative F_1_ sire families, we performed MASA to test whether the identified variants in the *MYH3* region fulfilled the biallelic QTL assumption [18, 19]. The results indicated that eight sires were heterozygous for the QTL genotype (*Q*/*q*) and four were homozygous (either *Q*/*Q* or *q*/*q*) (S2 Table and S7A Fig). Therefore, the genotype of the putative FSV should be homozygous in the four nonsegregating F_1_ sires and heterozygous in the eight segregating F_1_ sires in the LK cross. Application of this criterion to the 7,606 DSVs sorted out 548 putative FSVs in the 488.1-kb critical region in the LK cross. Likewise, this approach was applied to the F_1_ sires from the DK cross. We detected 5,211 DSVs in the critical region from the 5 DK F_1_ sires (S2 Table and S7B Fig). Among these variants, application of the same bi-allelic FSV criterion yielded 2,672 putative FSVs in the DK cross. Intersection of putative FSVs from the two crosses resulted in 547 overlapping putative FSVs.

**Fig 4:**
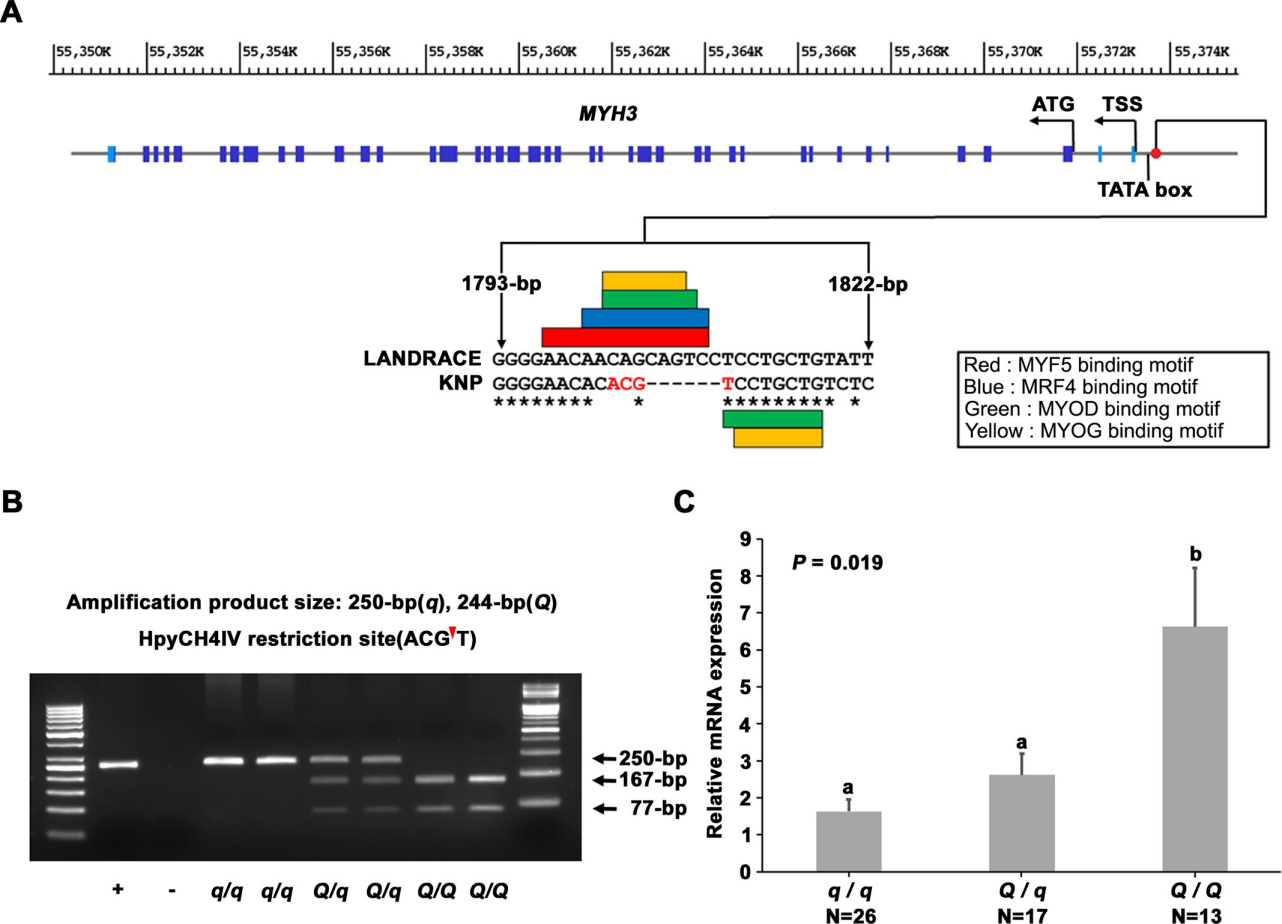
Genomic structure of porcine *MYH3*, its FSV, and the effect of the FSV on the *MYH3* expression. (**A**) The 2-kb region in the 5′- flanking region from the transcription start site (TSS), coding exons and introns, and the 0.5-kb region in the 3′- flanking region from the stop codon were Sanger sequenced. Light blue boxes represent 5′- flanking region and 3′- flanking region noncoding exons. The positions of the TATA box in the promoter and the TSS are indicated. Dark blue boxes represent coding exons, and the ATG initiation codon is designated. The red dot indicates the position of *XM_013981330*.*2*:*g*.*−1805_−1810del*. Predicted myogenesis regulatory factor (MRF) binding sites detected in the FSV sequence are presented in the box. (**B**) Determination of the genotype of the *XM_013981330*.*2*:*g*.*−1805_−1810del* was conducted by PCR amplification and subsequent *Hpy*CH4IV digestion. The *q*/*q* genotype represents the *MYH3* homozygous genotype originating from Landrace and Duroc pigs; the *Q*/*Q* genotype represents the *MYH3* homozygous genotype originating from KNPs. The + and–symbols represent positive and negative controls, respectively. (**C**) Messenger RNA expression levels for the porcine *MYH3* gene stratified by genotype at the *XM_013981330*.*2*:*g*.*−1805_−1810del* in *longissimus dorsi* muscle (least square mean±standard error). The significance of the effect of the FSV on gene expression was computed using the general linear model *y* = *μ*+*g*+*s*+*l*+*e*, where *y* is the relative mRNA expression level, *g* is the fixed effect of the genotype, *s* is the fixed effect of sex, *b* is the fixed effect of line, and *e* is residual. Different letters above the error bar show significant differences between genotypes (*P*<0.05).

To investigate whether these 547 variants are located in putative regulatory motifs in the noncoding regions of the 488.1-kb critical interval, we computationally predicted the motifs in the entire critical interval using the MEME suite [20]. Among the 547 variants, ninety putative FSVs were detected within the motifs predicted by the MEME suite (S3 Table). Subsequently, we investigated whether the ninety predicted motifs could be annotated using TRAP, JASPAR and PROMO programs [21–23]. A total of twenty-two predicted motifs were annotated as transcription factor binding sites based on the *in silico* analysis using the three programs (S3 Table). Notably, a motif located in the 2-kb of the 5′ promoter/regulatory region of the *MYH3* gene was predicted as the binding site for all four known myogenic regulatory factors (MRFs) (i.e., MYOD, MYOG, MYF5 and MRF4). The Sanger sequencing analysis of the 2-kb promoter region of the *MYH3* gene using the parental animals revealed a 6-bp deletion (*XM_013981330*.*2*:*g*.*−1805_−1810del*, chr12: 55,373,707) at the motif in the KNPs (Fig 4A). The 6-bp deletion variant, *XM_013981330*.*2*:*g*.*−1805_−1810del*, fulfilled the criteria of being a biallelic and overlapping FSV between the two cohorts. Moreover, this 6-bp deletion variant is located in the *MYH3* promoter at the position of the overlapping binding motifs for the four MRFs (Fig 4A). Thus, the *XM_013981330*.*2*:*g*.*−1805_−1810del* is expected to affect the binding of these MRFs.

### Bayesian fine-mapping of the 488.1-kb critical region characterizes *XM_013981330*.*2*:*g*.*−1805_−1810del* as a candidate causal FSV

To investigate whether the *XM_013981330*.*2*:*g*.*−1805_−1810del* obtained from the MASA and *in silico* functional annotation can be regarded as a candidate causal FSV, we applied Bayesian fine-mapping approaches using CAVIAR and eCAVIAR programs [24–26]. Prior to the fine-mapping analyses, we reconducted the PorcineSNP60K BeadChip-based GWAS including the *XM_013981330*.*2*:*g*.*−1805_−1810del* to obtain summary association statistics of the 488.1 critical region [27]. *XM_013981330*.*2*:*g*.*−1805_−1810del* was revealed as the top-ranked variants for a* and IMF in the 488.1-kb critical region (Table 1). In the Bayesian fine-mapping using the CAVIAR program, we established a 99% credible set of variants within which the candidate causal FSV(s) for a* and IMF are most likely to be included. The CAVIAR results revealed that the 99% credible set included only *XM_013981330*.*2*:*g*.*−1805_−1810del* with an extremely high posterior probability of being a candidate causal variant (S4 Table). Additionally, we performed bivariate Bayesian fine-mapping using the eCAVIAR program to provide evidence for whether *XM_013981330*.*2*:*g*.*−1805_−1810del* has a pleiotropic effect on a* and IMF. The combined likelihood posterior probability from eCAVIAR provided evidence that the *MYH3* 6-bp deletion variant can be considered as a pleiotropic variant for both a* and IMF (S4 Table).

**Table 1:** Effect of the *MYH3* functional sequence variant (FSV) on growth and meat quality traits of the *longissimus dorsi* muscle in LK and DK crosses (least square mean±standard error).

**Traits**^1^						
**LK cross**	***Q*/*Q***^2^ **(108)**	***Q*/*q*** **(427)**	***q*/*q*** **(568)**	***P-*value**		***P_Bonferroni_***^4^
Body weight at 140 d (kg)	67.23±0.98^a^	70.05±0.55^b^	72.52±0.48^c^	7.08±10^−8^		0.003
Carcass weight (kg)	72.93±1.21^a^	76.09±0.69^b^	79.14±0.59^c^	6.15×10^−7^		0.026
a* of LDM^3^ (AU)	2.42±0.02^a^	2.21±0.02^b^	1.88±0.01^c^	1.95×10^−70^		8.41×10^−66^
IMF of LDM^3^ (%)	1.72±0.04^a^	1.23±0.03^b^	0.67±0.03^c^	7.22×10^−89^		3.11×10^−84^
**DK cross**	***Q*/*Q*** **(54)**	***Q*/*q*** **(162)**	***q*/*q*** **(124)**	***P-*value**		***P_Bonferroni_***
Body weight at 140d (kg)	66.57±1.49	66.23±0.93	69.76±1.04	0.23		1
Carcass weight (kg)	65.95±1.63	67.99±1.03	71.15±1.16	0.016		1
a* of LDM (AU)	10.79±0.19^a^	8.76±0.12^b^	6.48±0.13^c^	4.18×10^−30^		8.51×10^−24^
Type1 fiber area^5^ (μm^2^)	378.3±163.2^a^	347.1±73.3^a^	292.0±79.7^b^	5.23×10^−9^		5.15×10^−5^
Type1 fiber areaP^6^ (%)	13.81±0.90^a^	11.23±0.38^a^	8.71±0.41^b^	9.64×10^−9^		7.22×10^−5^
IMF of LDM^3^(%)	2.17±0.06^a^	1.62±0.04^b^	0.96±0.04^c^	1.12×10^−3^^3^		3.38×10^−27^

^1^140d: 140 days of age; LDM: *longissimus dorsi* muscle; IMF: intramuscular fat content; AU: arbitrary unit.

^2^QTL genotypes correspond to the genotypes of the *XM_013981330*.*2*:*g*.*−1805_−1810del* variant and the number of pigs in each QTL genotype.

^3^Data that were natural log transformed.

^4^Probability of false positives per scan adjusted by Bonferroni’s method.

^5^Type 1 (slow/oxidative) fiber area and ^6^Type 1 (slow/oxidative) fiber area composition. Values with different superscripts (i.e., a,b and c) in a row are significantly different at the *P*<0.05 level.

These results provide information on the contrast between this particular 6-bp deletion variant and other variants used for GRAMMAR based GWAS. The regional *P*-value plot showed that it is extremely difficult to distinguish which variant should be considered as a candidate causal variant from the GRAMMAR based GWAS; there are several variants that can be regarded as candidate causal variants based on their *P*-value (S10 Fig). In this case, CAVIAR-based fine-mapping demonstrated a power to discriminate candidate causal variant(s) from the other variants in the GWAS.

The effect of this candidate causal variant on the phenotypes is shown in Table 1. The results show that the KNP originated *Q* allele is favorably associated with meat quality-related traits, whereas the Landrace originated *q* allele is positively associated with growth-related traits. The *Q* allele is also favorably associated with mRNA expression levels of the porcine *MYH3* gene in the *longissimus dorsi* muscle (Fig 4C). The genic action is mostly additive for all traits examined.

### Functional characterizations reinforce the critical importance of the *MYH3* 6-bp deletion variant

**Fig 5:**
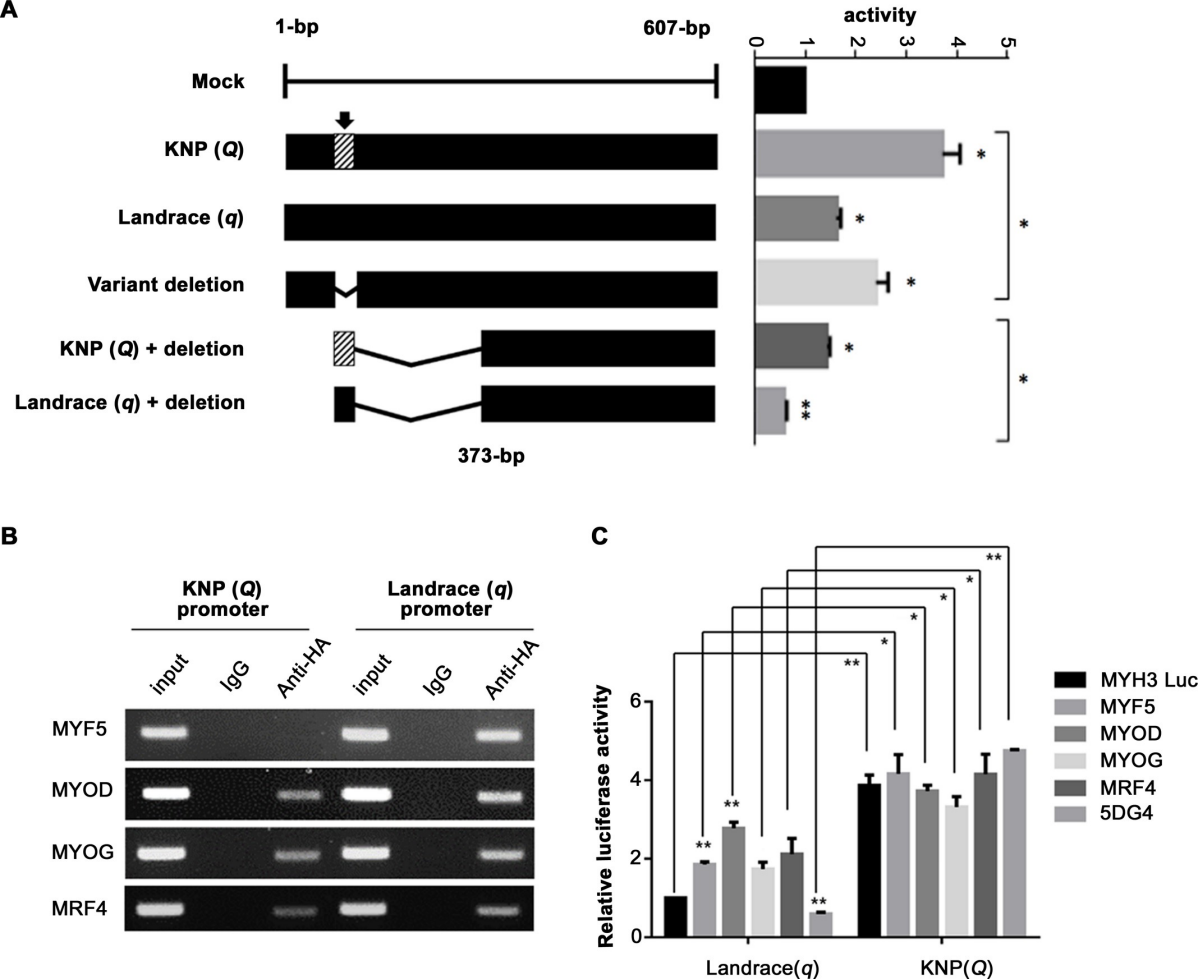
Analysis of promoter activity and transcription factor binding site in porcine fibroblast cells. (**A**) Schematic overview of the *XM_013981330*.*2*:*g*.*−1805_−1810del* luciferase reporter constructs and the results of the promoter activity assay. The arrow represents the sequence segment location of the *XM_013981330*.*2*:*g*.*−1805_−1810del*. Data histograms and error bars represent the mean±standard error of triplicate independent samples. *P<0.05, **P<0.01. (**B**) ChIP assay of the binding of MRFs to the *XM_013981330*.*2*:*g*.*−1805_−1810del* promoter region in porcine fibroblast cells derived from KNPs (*Q*) and Landrace (*q*). (**C**) Results from cotransfection experiments in porcine fibroblast cells. The *MYH3 q* promoter acts as a stronger repressor than the *MYH3 Q* promoter. The 5DG4 represents a cotransfection experiment with all four MRFs. Data histograms and error bars represent the mean±standard error of triplicate independent samples. *P<0.05, **P<0.01.

Subsequently, we analyzed the effect of the candidate functional variant of *MYH3* on transcription using transient transfection assays with luciferase reporter constructs containing either the Landrace (*q*) or KNP (*Q*) sequence fragments in porcine fibroblast cells [28, 29]. Compared with the promoterless construct, both the constructs containing the *Q* and the *q* sequence fragments increased luciferase reporter activity: the reporter activity increased ~3.7-fold for the *Q* construct and ~1.6-fold for the *q* construct. The reporter gene activity was consistently higher for the *Q* constructs than for the *q* constructs (Fig 5A). In addition, a chromatin immunoprecipitation (ChIP) assay of porcine fibroblast cells was conducted to investigate whether the four MRFs act as direct *trans*-acting factors that bind to the *XM_013981330*.*2*:*g*.*−1805_−1810del* site. The results of the ChIP assay demonstrated that MYF5 specifically bound to the *q* allele sequence, whereas the *Q* allele sequence that included the 6-bp deletion abolished its binding affinity. In the case of MYOD, MYOG, and MRF4, these MRFs were only able to interact partially with the *Q* sequence because the binding site was not completely abrogated by the 6-bp deletion (Fig 4A and Fig 5B).

To critically evaluate the functional effect of the four MRFs on the transcriptional activities of the luciferase reporter constructs containing either the *Q* or *q* sequence of the *MYH3* variant, we used transient cotransfection assays of porcine fibroblast cells. As shown in Fig 5C, when the *q* type of the *MYH3* promoter construct was cotransfected with either one of the four MRF constructs or the four MRFs, a significant reduction in luciferase activity was observed compared to the activity associated with the *Q* type. Notably, coexpression of the *q* type of the *MYH3* construct with all four MRFs led to significantly reduced luciferase activity compared with the activity in the fibroblast cells transfected with the empty control luciferase reporter alone. In contrast, overexpression of the four MRFs only weakly repressed the expression of the *Q* type of the *MYH3* reporter construct, which contains the 6-bp deletion. These data suggest that the *MYH3 q* variant is able to bind the four MRFs, whereas the *MYH3 Q* variant diminishes this interaction with their target sequence due to the less efficient binding of MRFs. Transient transfection assays with other combinations of MRFs showed the same trends regarding the expression activity of the luciferase reporter (S8 Fig). The results of transient transfection analyses indicate that the *MYH3 q* variant acts as a repressor element, whereas the *MYH3 Q* variant functions as a significantly weaker repressor, which results in overexpression of *MYH3* in the skeletal muscle of KNPs. Altogether, the functional characterization of *XM_013981330*.*2*:*g*.*−1805_−1810del* provided clear evidence that the porcine *MYH3* variant is a causal FSV affecting both a* and IMF.

### Allele frequency among pig populations indicates an Asian origin of the *MYH3* KNP allele

To investigate the *XM_013981330*.*2*:*g*.*−1805_−1810del*frequency across diverse pig breeds, we genotyped the *MYH3* FSV in a wide panel of 377 pigs representing nine Asian domestic breeds, 12 European domestic breeds, and wild boars from Africa, Europe, and Asia (S5 Table). The *Q* allele occurred at high and intermediate frequencies in Chinese Neijang (0.80), Chinese Putian (0.80), Chinese Tongcheng (0.90), Chinese Xiang (0.54), and KNP (0.63) but was also found in Korean and East Russian wild boars albeit at low frequency. In contrast, the *Q* allele was almost absent in the European commercial breeds, as well as in both European and African wild boars. This allele distribution indicates that the *MYH3 Q* allele is of Asian origin and likely predates domestication. Berkshire and Middle White breeds are an exception compared with the other European breeds. The rare exception for these breeds is probably due to an introgression of the Asian pigs in Europe, which was known to have occurred since the 18th century [30, 31]. Notably, we found that the *Q* allele is maintained at a moderate frequency despite its favorable effect on meat quality in KNPs. This could be due to the negative association of the *Q* allele with growth-related traits (Table 1). Sequence data indicated that nucleotide diversity in the critical region (12:55,073,130–55,561,243) was higher in Asian breeds (π = 0.0059) than in European breeds (π = 0.0038), which is consistent with the demographic history of pigs and in agreement with the results of previous studies (S6 Table) [32]. However, no decrease in the levels of genetic variability surrounding the *MYH3* FSV was observed in the European breeds compared to the levels in the Asian breeds. Although no signature of selection was detected with Tajima's D statistics, an interesting possible exception was KNPs, for which Tajima’s D = 2.50 at the promoter region may indicate balancing selection. Furthermore, we observed no reduction in diversity in the *MYH3* and its promoter region because of domestication, neither in Europe nor in Asia. The absence of a significant selective footprint (as measured by Tajima’s D or low diversity) in breeds with high frequency of the *Q* allele may be due to soft sweeps, which are much more difficult to detect than hard sweeps with these tests. In addition, Tajima’s D is highly variable in the presence of ongoing selection [33].

## Discussion

Although numerous genetic studies have mapped thousands of QTLs for complex quantitative traits [34], a very limited number of actual causative mutations have been detected in domesticated animals. This study is the first to show that *MYH3* is a causative gene for myofiber type ratios and associated changes in IMF and adipogenesis in pigs and mice. *MYH3* is known as an MYH isoform that is mainly expressed in various developmental stages including during the embryonic stage in skeletal muscle [35, 36]. Additionally, coding mutations that occur in the *MYH3* gene can cause muscle development disorders in humans [37, 38]. Furthermore, we identified a structural variant in the promoter of the *MYH3* gene that affects both muscle fiber-type specification and IMF accumulation using two independent crosses in pigs. We also discovered that the FSV alters the sequence of critical transcription factor binding sites located in the promoter of *MYH3*. Subsequently, we showed that the FSV of *MYH3* can regulate transcription by differential binding of the four MRFs using a chromatin immunoprecipitation (ChIP) assay and transient transfection experiments in porcine fibroblast cells.

As shown in Table 1, the causative FSV was also related to growth traits in pigs. However, there was no significant body weight difference observed between transgenic and wild-type mice. A plausible interpretation of this result can be related to pleiotropy and linkage; if the FSV has a pleiotropic effect on both meat quality and growth traits, a body weight difference between transgenic and wild-type mice can be expected. Hence, the observed association does not necessarily indicate pleiotropy (i.e., the growth and meat quality related traits are influenced by the same causal variant), since the association could possibly be due to linkage (i.e., two different causal variants being in linkage disequilibrium, one influencing meat quality related traits and the other influencing growth related traits). Hence, it is possible that the variant affecting growth variation may not be included in the transgenic vector construction. This antagonistic effect of the FSV between the favorable meat quality traits and growth parameters can be concerned when this variant is considered in the implementation of current breeding programs. However, we are convinced that the *MYH3* variant can be useful for inclusion in breeding programs; if a marker-assisted selection/gene editing strategy using this variant can be implemented together with traditional selection procedures, we can expect to obtain genetic gain in growth traits in a long-term perspective. In fact, we previously reported growth-related QTLs in the LK cross, but no major QTL was detected **[39]**. Given the polygenic architecture of growth, we argue that growth traits can also be improved by a combined approach using the *MYH3* marker information and traditional selective breeding.

The moderate to high frequency of the *Q* allele among some of the Asian domestic pig breeds including KNPs along with the positive association of the *q* allele with growth performance indicate that some sort of balancing selection may have shaped the evolution of *MYH3* in pigs. This case is similar to the case of the porcine *RYR1* gene, which causes pale soft exudative meat and porcine stress syndromes in homozygotes but has a positive effects on muscle mass in heterozygotes [40]. An additional argument in favor of balancing selection could be the positive Tajima’s D value in KNPs and the absence of a decrease in variability in European pigs (S6 Table); however, the exact mechanism remains to be elucidated since the *Q* allele is almost absent in European pigs (S5 Table).

In conclusion, we present the positional cloning of porcine *MYH3* as a QTG and the identification of a 6-bp deletion FSV located in the 5′-flanking region that regulates transcription of the *MYH3* gene and contributes to a major effect on both a* and IMF in pigs. This work enhances the understanding of the regulation of myotype ratios and associated changes in IMF in pig skeletal muscle. Furthermore, this information is immediately applicable for breeders who are actively involved in genetic improvement of pork quality through marker-assisted selection or introgression of the desired structural variant allele in breeding populations.

## Materials and methods

### Ethics statement

All experimental procedures using pigs were conducted according to national and institutional guidelines and were approved by the Ethical Committee of the institution [Approval No. (date): 2014–095 (2014-08-06)]. All mouse experiments were approved by the Institutional Animal Care and Use Committee of the KRIBB [Approval No. (date): KRIBB-AEC-16077 (2016-04-01)] and were performed in accordance with the Guide for the Care and Use of Laboratory Animals published by the U.S. National Institutes of Health.

### Animals and phenotypes

Two independent cohorts were used in this study: a Landrace×KNP F_2_ intercross and a Duroc×KNP F_2_ intercross. The first cross was established as described previously [7]. Briefly, seventeen purebred Landrace pigs were mated with 19 purebred KNPs to produce a total of 91 F_1_ progeny and 1,105 F_2_ progeny (568 males and 537 females). For the second cross, nine purebred Duroc pigs were crossed with 5 purebred KNPs to produce 36 F_1_ and 345 F_2_ animals (187 males, 158 females). Animals in the two cohorts were raised at the experimental farm of the National Institute of Animal Science, Jeju, Republic of Korea. They were fed *ad libitum*, and males were not castrated. All F_2_ experimental animals were slaughtered in the same commercial slaughterhouse. The means and standard errors of age at slaughter (days) were 199.2±0.35 (LK cross) and 195.7±0.66 (DK cross). The means and standard errors of carcass weight (kg) were 79.2±0.38 (LK cross) and 69.7±0.68 (DK cross). The approximate average slaughter ages of the pigs used for expression analysis were 170 (Landrace) and 180 (KNP) days. The data collection of meat quality-related traits (i.e., a* and IMF) was conducted as reported previously [8]. Traits related to muscle fiber characteristics were obtained using the myofibrillar ATPase staining method [41] and microscope counting.

### Genotypes and genome-wide association study (GWAS)

All experimental samples were genotyped for 62,163 SNP markers using PorcineSNP60K BeadChip (Illumina). The SNP markers were filtered with a minor allele frequency <5%, a genotype call rate <90% and a *P*-value of the Chi-square test for Hardy-Weinberg equilibrium errors ≤0.000001. For the LK cross, a total of 40,628 SNPs on 18 autosomes remained for this GWAS. For the DK cross, a total of 39,964 SNPs on 18 autosomes remained for this GWAS. Error rates in Mendelian inheritance for all markers were also checked in the F_2_ pedigrees using SNP marker information (<5%). A single-SNP marker analysis based on the genome-wide rapid association using a mixed model and regression (GRAMMAR) approach was carried out to identify QTLs affecting meat quality-related traits in the two cohorts [42]. Each trait was adjusted for fixed (sex, batch and carcass weight) and random polygenic effects using the mixed-effects model method implemented in the ASReml program (VSN International). To estimate random polygenic effects, the kinship matrix computed from the F_2_ pedigree was used. The residuals derived from the mixed-effects model were used as response variables in the linear regression analyses to correct for the familial relatedness within the F_2_ intercross population. The GWA analyses were conducted under an additive model for each SNP using the PLINK program [43]. Bonferroni-adjusted significant (i.e., 0.05/40,628 markers, significant *P*-value = 1.23×10^−6^ for the LK cross; 0.05/39,964 markers, significant *P*-value = 1.25×10^−6^ for the DK cross) and suggestive (i.e., 1/40,628 markers, suggestive *P*-value = 2.46×10^−5^ for the LK cross; 1/39,964 markers, suggestive *P*-value = 2.50×10^−5^ for the DK cross) thresholds were established to address multiple testing issues in GWAS. The % phenotypic variance explained by the top-ranked SNP marker was (% Var_SNP_) estimated using the following equation [44]: % Var_QTL_ = [2*p*(1-*p*)a^2^ / σ_g_^2^] ×100, where *p* is the minor allele frequency of the SNP, a is the estimated allelic effect of the SNP, and σ_g_^2^is the additive genetic variance for each trait estimated by the ASReml program (VSN international). The % phenotypic variance explained by the QTL was (% Var_QTL_) estimated by the following equation: [(RSS_reduced_−RSS_full_)/RSS_reduced_] × 100, where RSS_reduced_ and RSS_full_ are residual sum of squares of statistical models of linkage analysis with and without the QTL term, respectively.

### Joint linkage and linkage disequilibrium (LALD) analysis

High-resolution mapping of the QTL was conducted by jointly exploiting linkage and linkage disequilibrium using a haplotype-based approach. First, we used CRI-MAP ver 2.503, developed by Evans and Maddox (URL: www.animalgenome.org/bioinfo/tools/share/crimap), to construct the genetic linkage map of SSC12 using 935 SNP markers for the Landrace×KNP cross and 997 SNP markers for the Duroc×KNP cross. Second, founder haplotypes those found in the F_0_ pigs were reconstructed using the DualPHASE program [13], which combines family (linkage and Mendelian segregation) and population (linkage disequilibrium) information in a Hidden Markov Model frame. A total of twenty founder haplotype clusters (*K* = 20) were used for the next step. Third, the haplotypes were incorporated into a mixed model including fixed (sex, batch and carcass weight), random (i.e., the twenty effects of founder haplotypes and animal effects), and random residual terms to perform fine mapping of QTL using the QxPAK ver 5.05 [45]. In addition, the LALD mapping with the effect of the most likely QTL position as cofactor was conducted to screen additional QTL signals in the chromosome of interest.

### Marker-assisted segregation analysis (MASA) and *in silico* functional annotation in the 488.1-kb critical region on SSC12

MASA was performed in two ways: first, we conducted a half-sib QTL analysis to obtain evidence for QTL segregation within each F_1_ sire-family. Identification of heterozygous *Q*/*q* F_1_ sires was based on the results of the half-sib QTL analysis using their respective F_2_ progeny (S7 Fig) [18]. Second, the QTL genotype of F_1_ sires was determined by a log likelihood ratio test [19]. The F_1_ sires were regarded as heterozygous if log_10_likelihood ratio score>2; homozygous if log_10_likelihood ratio score>−2; and undetermined genotype when −2 < log_10_likelihood ratio < 2 (S2 Table). The transcription start site of the *MYH3* gene was identified using Promoter 2.0 [46]. To investigate whether the identified putative FSVs were located within transcriptional regulatory motifs, the MEME suite was used [20]. Subsequently, a detailed *in silico* annotation of the identified motifs was conducted by TRAP, JASPAR and PROMO programs, respectively [21–23]. These four programs were used with their default settings.

### Genotype imputation and fine-mapping of the 488.1-kb critical region

Prior to the fine-mapping approaches, we imputed genotype data in the critical region using FImpute program which can utilize information from both pedigrees and populations [47]. The genotype imputation was conducted with the default settings of the FImpute program. For the imputation, we only used the variants from the 60K SNP chip and the 6-bp deletion variant for further fine mapping to fill in missing genotypes of the variants. To characterize the identified putative FSV in the 488.1-kb critical region, a Bayesian fine-mapping approach based on the CAusal Variants Identification in Associated Regions (CAVIAR) program [25]. The CAVIAR program incorporates association summary statistics (i.e., Z-scores) and LD correlation structures to compute the posterior probability of being candidate causal for each variant in the region of fine-mapping. The eCAVIAR program [26], a bivariate extension of CAVIAR, was also applied to fine-map the causal variant by colocalization analysis of association signals from a* and IMF. eCAVIAR calculates combined likelihood posterior probability (CLPP) to measure the degree of colocalization of the two QTLs by computing the probability that the variant is pleiotropic for both phenotypes. A threshold of 0.99 for both posterior probability and CLPP was applied to select candidate causal variants in the CAVIAR and eCAVIAR analyses. To evaluate the uncertainty of the fine-mapping analyses, 99% credible sets were constructed for both CAVIAR and eCAVIAR analyses (S4 Table).

### Gene transcription analysis

Total RNA was isolated from cells and tissues using Trizol reagent (Ambion), according to the manufacturer’s protocol. RNA was treated with DNase I and reverse transcribed into cDNA using the TOPscript cDNA Synthesis Kit (Enzynomics). For cDNA synthesis, 5 μg of each sample RNA was incubated at 55°C for 60 min and at 95°C for 5 min. Each cDNA was used as a template for qRT-PCR amplification in combination with specific primers (S7 and S8 Tables). We performed qRT-PCR using the QuantiTect SYBR Green PCR Kit (Qiagen) and the Rotor-Gene Q thermal cycler (Qiagen). The qRT-PCR experiments were conducted based on the MIQE guidelines [48]. qRT-PCR was performed for 40 cycles at 95°C for 20 sec, at 60°C for 20 sec, and at 72°C for 20 sec. Transcription levels were normalized to those of *GAPDH* mRNA. *GAPDH* has been used as the reference gene in several expression studies related to myogenesis and adipogenesis [49–52]. Data were analyzed using the ΔΔC_t_ method [53].

### NGS and Sanger sequencing

A massively parallel sequencing technology was used to identify SNP markers in the porcine whole genome, including the *MYH3* locus on SSC12, using genomic DNA of the 24 F_1_ sires and the parental pigs [Landrace (n = 17) and KNPs (n = 19) for the LK cross; Duroc (n = 9) and KNPs (n = 5) for the DK cross] from the two crosses (i.e., 18 pigs from the LK cross; 6 pigs from the DK cross). Adapter-ligated DNA libraries were prepared for single and paired-end sequencing. All sequence data were produced using HiSeq X (Illumina) according to the standard protocol. The average coverage depth was approximately 30×. In addition, the Sanger sequencing method using BigDye Terminator 134 v3.1 Cycle Sequencing Kit (Applied Biosystems) was applied to determine the sequence of the 5' and 3' flanking regions and exon regions of the porcine *MYH3* gene using DNA samples from the parental animals of the two cohorts [i.e., Landrace (n = 17) and KNPs (n = 19) for the LK cross; Duroc (n = 9) and KNPs (n = 5) for the DK cross].

### Generation of transgenic (TG) mice

All mice (C57BL/6 background, male, 4 months of age) used in the study were maintained in the Korea Research Institute of Bioscience and Biology (KRIBB) animal facility under pathogen-free conditions in a temperature-controlled climate at 22±2°C and with a 12 h light/dark cycle. All animals had free access to standard chow and water during the experiments. For the convenience of cloning, the CDS of the porcine *MYH3* gene was divided into four parts for *in vitro* synthesis (Bioneer). We lined them up end-to-end to make a 5,859 bp full-length open reading frame (ORF) and inserted it into the position between the *Xba*I and *EcoR*I of a pCAGGS-EGFP-Puro vector (S6A Fig). In addition, a Flag-sequence was appended to the 3′ end of the ORF to allow for the detection of the expression of recombinant protein directly through Western blotting. TG-mice were generated via DNA microinjection. PCR analysis was applied to test the construction of TG-mice using a primer set (forward: 5′- CCG AGA GCT GGA GTT TGA-3′; reverse: 5′- CTC CCA TAT GTC CTT CCG AGT-3′). Quadriceps muscles were excised from 4-month old WT- and TG-mice and used for further experiments.

### Western blotting analysis

Tissue protein lysates were prepared with RIPA buffer containing a cocktail of protease inhibitors and then quantified using a Protein Assay Kit (Bio-Rad). Protein samples were separated by SDS-PAGE and transferred to a PVDF membrane. After blocking in TBST solution containing 5% skim milk, membranes were incubated overnight at 4°C with specific antibodies (S9 Table). Expression signals of each protein were detected using ECL reagents (GE Health Care) with secondary antibodies. Luminescent densities were measured using a LAS-3000 Luminescent Image Analyzer System (Fujifilm).

### Histological analysis of mice

Traits related to muscle fiber characteristics were obtained using the myofibrillar ATPase staining method described by Brooke and Kaiser (1970) and microscope counting. For IHC analysis, paraffin sections (4 μm) were fixed in 4% PFA, permeabilized with 0.1% Triton X-100 and left for 1 h in serum containing blocking solution. Anti-MYH7, anti-MYH4 and anti-flag M2 antibodies were diluted to 1:100 in PBS and incubated overnight at 4°C. After washing with PBS containing Tween 20, the sections were incubated for 1 h with fluorescent conjugated secondary antibodies and visualized under a fluorescence microscope. DAPI was used for nuclei counterstaining.

### Measurement of triacylglycerol (TAG), triglycerides and free fatty acids (FFAs)

Both WT and TG mice were fasted for 6 h before sacrifice, and dissected *quadriceps* muscle and blood were obtained. TAG, triglycerides and FFAs were assayed using a Triglyceride Assay Kit (ABCAM) and a Free Fatty Acid Assay Kit (ABCAM). The levels of TAG, triglycerides, and FFAs were measured by the fluorescent intensities at an absorbance ratio of 535/590 nm wavelength. Finally, the levels of TAG, triglycerides, and FFAs were calculated based on typical standard curves.

### Luciferase and chromatin immunoprecipitation (ChIP) assays

Primarily cultured porcine fibroblast cells, originating from biopsied porcine ear tissues, were cultured in DMEM (Life Technologies) containing 10% FBS (HyClone) and 1×penicillin-streptomycin reagent (Gibco). Cells were grown at 37°C in humidified air containing 5% CO_2_ and the medium was changed every 2 days. The porcine fibroblast cells have been used for our other studies [28, 29]. All *MYH3* luciferase reporter constructs were generated by subcloning the porcine *MYH3* promoter in front of the luciferase gene in the pGL3 basic vector. The *MYH3* luciferase reporter constructs and internal control (pRL-SV40) vectors were cotransfected into the cells using Lipofectamine 2000 (Invitrogen). Luciferase assays were performed using a Dual-Luciferase Reporter Assay System (Promega). Transfected cells were rinsed in PBS and then lysed in 1× passive lysis buffer, after measuring firefly (reporter construct) and Renilla (internal control) luciferase values using the VICTOR Multilabel Plate Reader (Perkin Elmer). MRF genes (i.e., *MYF5*, *MYOD*, *MYOG*, and *MRF4*) were cotransfected with an *MYH3* promoter and an internal control vector into porcine fibroblast cells. After 48 h, *MYH3* promoter activity was measured using the Dual-Luciferase Reporter Assay System. The influence of *MYH3* promoter activity was determined according to MRFs binding in the functional sequence variant position. ChIP assays were carried out using a Chromatin Immunoprecipitation Assay Kit (Millipore). Precleared chromatin was immunoprecipitated with 4 μg of the HA antibody (Sigma). The obtained DNA samples were verified by PCR analysis (forward 5′-GGT CCT ACT GGC GCT TAA GAC AGA-3′; reverse 5′- GGT TGT GGC AGG AAT GTG TGA TTG-3′) for the MRFs binding motif on the *MYH3* promoter region.

### Statistical analysis

The results are expressed as the mean±standard error. Minitab version 17 (Minitab Inc.) and MS Excel (Microsoft Inc.) were used to evaluate statistical significance using two-sided Student’s t-test or two-sided Welch’s t-test when comparing two groups or analysis of variance for multiple group comparisons. The *F*-test implemented in the Minitab program was applied to assess variance equality between two groups (*P*-value 0.01). *P*<0.05 was considered as significant for the two-sample t-tests. Unless otherwise stated *P*-values are nominal; the nominal *P*-value represents the probability of false positives for a single test.

### Population genetic analysis

The *XM_013981330*.*2*:*g*.*−1805_−1810del* was genotyped in 377 wild and domestic pigs distributed worldwide (S5 Table). Furthermore, to characterize the genetic variability surrounding the region, a total of 160 NGS data samples were analyzed (S6 Table); 65 sequences were downloaded from SRA [54–58]; and 10 KNP, 10 Duroc, and 10 Landrace NGS data were obtained in this study. The sequences included wild boars (10 Korean and 10 of European origin) and domestic pigs (34 Chinese and Korean and 41 European). The Chinese pig breeds were Meishan and Toncheng, and the European pig breeds were Berkshire, Duroc, Iberian, Landrace, and Large White. Downloaded samples from SRA had been sequenced to an average depth of approximately 11×, whereas the depth of those in this study (i.e., KNPs, Duroc and Landrace) was approximately 30×. Alignment was carried out with BWA 0.7.15—mem option [59]. The bam files were then realigned around indels with the GATK IndelRealigner tool [60], and PCR duplicates were removed with the picard MarkDuplicates option (http://broadinstitute.github.io/picard/). SNP calling was performed with SAMTOOLS/BCFTOOLS suite version 1.3.1 for each individual separately [61]. SNPs were called in positions with depth bounds between 5× and twice the average depth rounded to the nearest upward integer; further, a minimum mapping (RMS) quality of 20 and a base quality of 20 were required. Finally, SNPs with a minimum quality of 10 were retained. To estimate nucleotide diversity, in addition to heterozygous positions, the number of bases sequenced was also required. We extracted these regions with minimum and maximum depth using SAMTOOLS depth, further filtering by minimum map and base qualities and then, using BEDTOOLS [62], we intersected these regions with the homozygous blocks provided in the individual gvcf file. This resulted in a modified gvcf file in which both SNPs and homozygous blocks had been filtered by the same criteria. Diversity and Tajima’s D estimates were as in accounting for missing data [63], and as implemented in MSTATPOP software [64].

## Supporting information

S1 Fig Manhattan plots and quantile–quantile plots of the GWAS for a* and IMF traits in LK and DK crosses. The y-axis shows the −log_10_*P*, and the x-axis shows the physical positions of the SNP markers on the pig autosomes. The genome-wide significant threshold value is 5.90, equals Bonferroni’s correction of 5% (represented by the red horizontal lines). (**A**) For a* in the LK cross (n = 963); (**B**) For IMF in the LK cross (n = 962); (**C**) For a* in the DK cross (n = 294); (**D**) For IMF in the DK cross (n = 294). The Manhattan plots show the identification of the major QTL for a* and IMF traits on SSC12 in the two crosses. The genomic inflation factor (λ) was 1.0 for all four results of GWAS.
(TIF)

S2 Fig Linkage mapping of a QTL that influences a* and IMF contents in the *longissimus dorsi* muscle of the LK (n = 1,232) and DK (n = 395) crosses. (**A**) Linkage mapping results on SSC12 for a* and IMF traits in the LK cross (**B**) Linkage mapping results on SSC12 for a* and IMF traits in the DK cross. The y-axis represents the *F*-value test statistic. The marker map with genetic distance between DNA markers in Kosambi cM is given on the x-axis. The thick horizontal line indicates the 1% chromosome-wide significant threshold, and the thin horizontal line indicates the 5% chromosome-wide significant threshold. The QTLs were colocalized in the region encompassing rs81437379. Linkage analysis for mapping QTL was performed using the GridQTL program (URL:www.gridqtl.org.uk).
(TIF)

S3 Fig Cross-specific and shared critical regions on SSC12 identified by joint linkage and association mapping. SNP position is the physical base pair position in SSC12 (Sus scrofa 11.1). LOD_a* and LOD_IMF represent the LOD (logarithm of odds) score for the redness meat color and intramuscular fat content. The red-colored values represent maximum LOD scores for a* and IMF traits in each intercross. The dark gray region represents the critical region (12:54,842,795–55,561,243) for the LK cross, while the light gray region (12:55,073,130–55,931,714) indicates the critical region for the DK cross. A conservative 2-LOD drop support interval was applied to estimate the critical region. The black box line is highlights the 488.1-kb shared critical region. Numbers in the ovals represent the LOD drop support.
(TIF)

S4 Fig A Neighbor-joining phylogenetic tree for mammalian *MYH* genes, based on genetic distances computed with Kimura’s two-parameter method, was constructed using MEGA7 (URL: www.megasoftware.net). Multiple sequence alignment was performed with DIALIGN2.2.1 (URL:dialign.gobics.de). Numbers at the nodes represent the bootstrap support values derived from 10,000 replicates. The scale indicates the genetic distance. The accession numbers for the mRNA sequences are provided in Table S1. The species used are as follows: *Bo* (cattle), *Ch* (green monkey), *Ca* (dog), *Go* (Gorilla), *Ho* (human), *Mu* (mouse), *Ra* (rat), and *Su* (pig). We used the *Dr* (fruit fly) *MYH* mRNA sequence (NM_165190.4) as the out group. The *MYH* isoforms formed distinct clusters and this result provided conclusive evidence that *MYH13* (*LOC100736982*) and *MYH8* (*LOC110255887*) have been identified in pigs by this analysis.
(TIF)

S5 Fig Estimated effect of founder haplotypes on a* and IMF and frequencies of in the two studied populations. Founder haplotypes showing similar effects were pooled. Founder haplotypes associated with the inferred *q* and *Q* alleles of the later-detected as candidate functional sequence variants are shown in blue and red boxes, respectively. Phenotype data in the three panels (**A, C** and **D**) were natural log transformed. (**A, C**) For LK cross; (**B, D**) For DK cross.
(TIF)

S6 Fig Identification and characterization of TG-mice. (**A**) Transgenic construction of the porcine *MYH3* vector. The construct consists of the CAG promoter, porcine *MYH3* mRNA sequence, flag for protein detection and pA (poly A) (upper panel). Western blotting analysis revealed that the 24 F_1_ founder showed the highest expression of MYH3 protein. The x-axis represents TG-mouse id. (**B**) Estimated porcine *MYH3* transgene copy number in each TG. The x-axis represents TG-mouse id. The porcine *MYH3* copy number ranged from 2 to 13 in each TG-mouse. (**C**) Body weight comparison between WT (n = 3) and TG (n = 4) mice. Body weights of male mice were measured at 4 months of age. (**D**) Comparison of the area of slow (type1/oxidative) and fast (type2) muscle fibers between WT (n = 5) and TG (n = 5) mice. The horizontal bars indicate median. (**E**) Expression of slow and fast muscle-associated genes in *quadriceps* muscle. Analyses of slow-type (left) and fast-type (right) muscle- associated gene expression by qRT-PCR. Four-month-old WT (n = 3) and TG (n = 4) mice were used. Data are mean±standard error for three independent replicates. *P<0.05, **P<0.01.
(TIF)

S7 Fig Identification of informative segregating F_1_ sires. Maximum chromosome-wide −log_10_*P* values for a* and IMF in each of the analyzed half-sib sire families by using the GridQTL program (URL:www.gridqtl.org.uk). (**A**) Twelve for the LK cross; (**B**) Five for the DK cross. The chromosome-wide significance levels (1% for *A*; 5% for *B*) obtained from 10,000 permutations are shown as horizontal lines. Numbers above the bar graph correspond to the most likely chromosome position of QTL (cM). The numbers in the parentheses represent the number of progeny in each sire family. The black triangles indicate the sire families segregating for QTL.
(TIF)

S8 Fig Analysis of luciferase assay using the *MYH3* promoter with various MRF combinations. Reporter and MRFs constructs were electroporated in porcine fibroblast cells (*MYH3* Luc, ‘empty’ vector cotransfected with MRF constructs). Luciferase activity of KNP (*Q*) was compared with that of Landrace (*q*). The *MYH3* KNP (*Q*) promoter acts as a weaker repressor than the *MYH3* Landrace (*q*) promoter. Data histograms and error bars represent the mean±standard error of three independent samples. ***P<0.001.
(TIF)

S9 Fig Full blot figures. **(A)** Full blot figures of Fig 2E; **(B)** Full blot figures of Fig 3C; **(C)** Full blot figures of Fig 3D.
(TIF)

S10 Fig Regional *P*-value plots obtained from GRAMMAR based GWAS for a* and IMF. The y-axis shows the −log_10_(*P*-value), and the x-axis shows the physical positions of the SNP markers on the pig chromosome 12. The genome-wide significance threshold value is 5.90, which equals Bonferroni’s correction of 5% (represented by the red horizontal lines). There are lines to connect the pairwise LD structure with a black horizontal line representing the 488.1-kb critical region. The physical position of each SNP marker is demonstrated above the LD plot. The * indicates the position of *MYH3* FSV. The magnitude of LD by r-square statistic is shown. (**A**) For a* in the LK cross (n = 963); (**B**) For IMF in the LK cross (n = 962).
(TIF)

S1 Table Messenger RNA sequence identification, Refseq name, and physical position used for the phylogenetic analysis. (DOCX)

S2 Table Determination of QTL genotypes of F1 sires by marker assisted segregation analysis in LK and DK crosses (mean±standard error). (DOCX)

S3 Table Positions of overlapped putative FSVs located in predicted regulatory motifs in the 488.1-kb critical region. (DOCX)

S4 Table Results of CAVIAR and eCAVIAR analyses using the Porcine60K BeadChip chip variants in the 488.1-kb critical region. (DOCX)

S5 Table Allele frequency of the *MYH3* FSV among pig populations. (DOCX)

S6 Table Nucleotide diversities per base pair and Tajima's D statistics by region and by pig population, obtained from re-sequencing data. (DOCX)

S7 Table qRT-PCR primers for analysis of mouse muscle samples. (DOCX)

S8 Table qRT-PCR primers for analysis of muscle samples from pigs. (DOCX)

S9 Table List of antibodies used in this study. (DOCX)

S10 Table Resequencing data access information. (DOCX)
