# Correction: A functional regulatory variant of *MYH3* influences muscle fiber-type composition and intramuscular fat content in pigs

**DOI:** 10.1371/journal.pgen.1011715

**Published:** 2025-05-20

**Authors:** In-Cheol Cho, Hee-Bok Park, Jin Seop Ahn, Sang-Hyun Han, Jae-Bong Lee, Hyun-Tae Lim, Chae-Kyoung Yoo, Eun-Ji Jung, Dong-Hwan Kim, Wu-Sheng Sun, Yuliaxis Ramayo-Caldas, Sang-Geum Kim, Yong-Jun Kang, Yoo-Kyung Kim, Hyun-Sook Shin, Pil-Nam Seong, In-Sul Hwang, Beom-Young Park, Seongsoo Hwang, Sung-Soo Lee, Youn-Chul Ryu, Jun-Heon Lee, Moon-Suck Ko, Kichoon Lee, Göran Andersson, Miguel Pérez-Enciso, Jeong-Woong Lee

The hyperlink in the Data Availability statement is incorrect. The correct hyperlink is: https://datadryad.org/dataset/doi:10.5061/dryad.dr32n87.

## References

[1] Cho I-C, Park H-B, Ahn JS, Han S-H, Lee J-B, Lim H-T, et al. A functional regulatory variant of MYH3 influences muscle fiber-type composition and intramuscular fat content in pigs. PLoS Genet. 2019;15(10):e1008279. doi: 10.1371/journal.pgen.1008279 31603892 PMC6788688

