# Supplementary figures and images for: A functional regulatory variant of MYH3 influences muscle fiber-type composition and intramuscular fat content in pigs
Source: PLoS Genet. 2019 Oct 11;15(10):e1008279. doi: 10.1371/journal.pgen.1008279 (PMC6788688; doi:10.1371/journal.pgen.1008279)

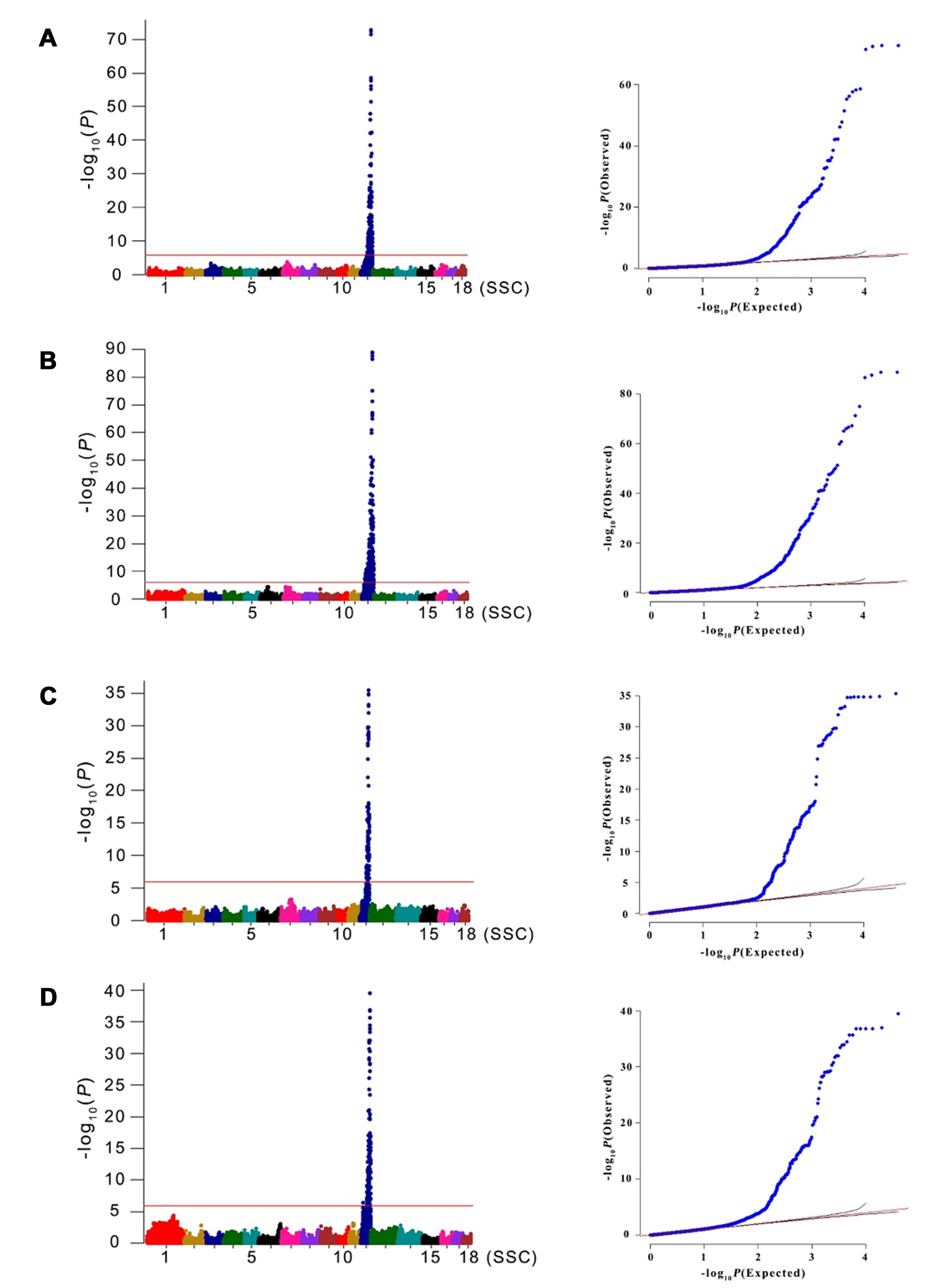

Supplement: S1 Fig — The y-axis shows the −log10P, and the x-axis shows the physical positions of the SNP markers on the pig autosomes. The genome-wide significant threshold value is 5.90, equals Bonferroni’s correction of 5% (represented by the red horizontal lines). (A) For a* in the LK cross (n = 963); (B) For IMF in the LK cross (n = 962); (C) For a* in the DK cross (n = 294); (D) For IMF in the DK cross (n = 294). The Manhattan plots show the identification of the major QTL for a* and IMF traits on SSC12 in the two crosses. The genomic inflation factor (λ) was 1.0 for all four results of GWAS. (TIF) [file pgen.1008279.s001.tif]

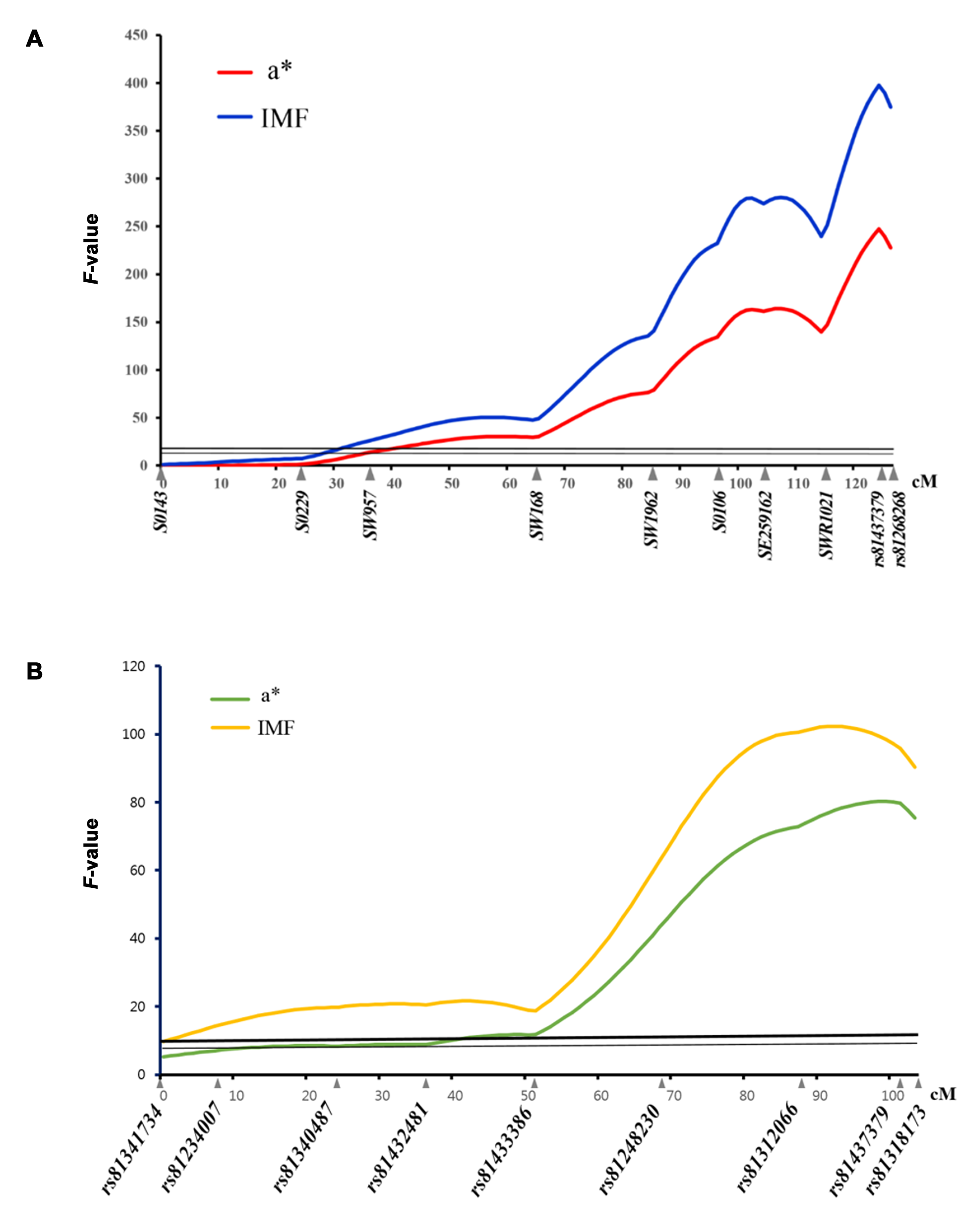

Supplement: S2 Fig — (A) Linkage mapping results on SSC12 for a* and IMF traits in the LK cross (B) Linkage mapping results on SSC12 for a* and IMF traits in the DK cross. The y-axis represents the F-value test statistic. The marker map with genetic distance between DNA markers in Kosambi cM is given on the x-axis. The thick horizontal line indicates the 1% chromosome-wide significant threshold, and the thin horizontal line indicates the 5% chromosome-wide significant threshold. The QTLs were colocalized in the region encompassing rs81437379. Linkage analysis for mapping QTL was performed using the GridQTL program (URL:www.gridqtl.org.uk). (TIF) [file pgen.1008279.s002.tif]

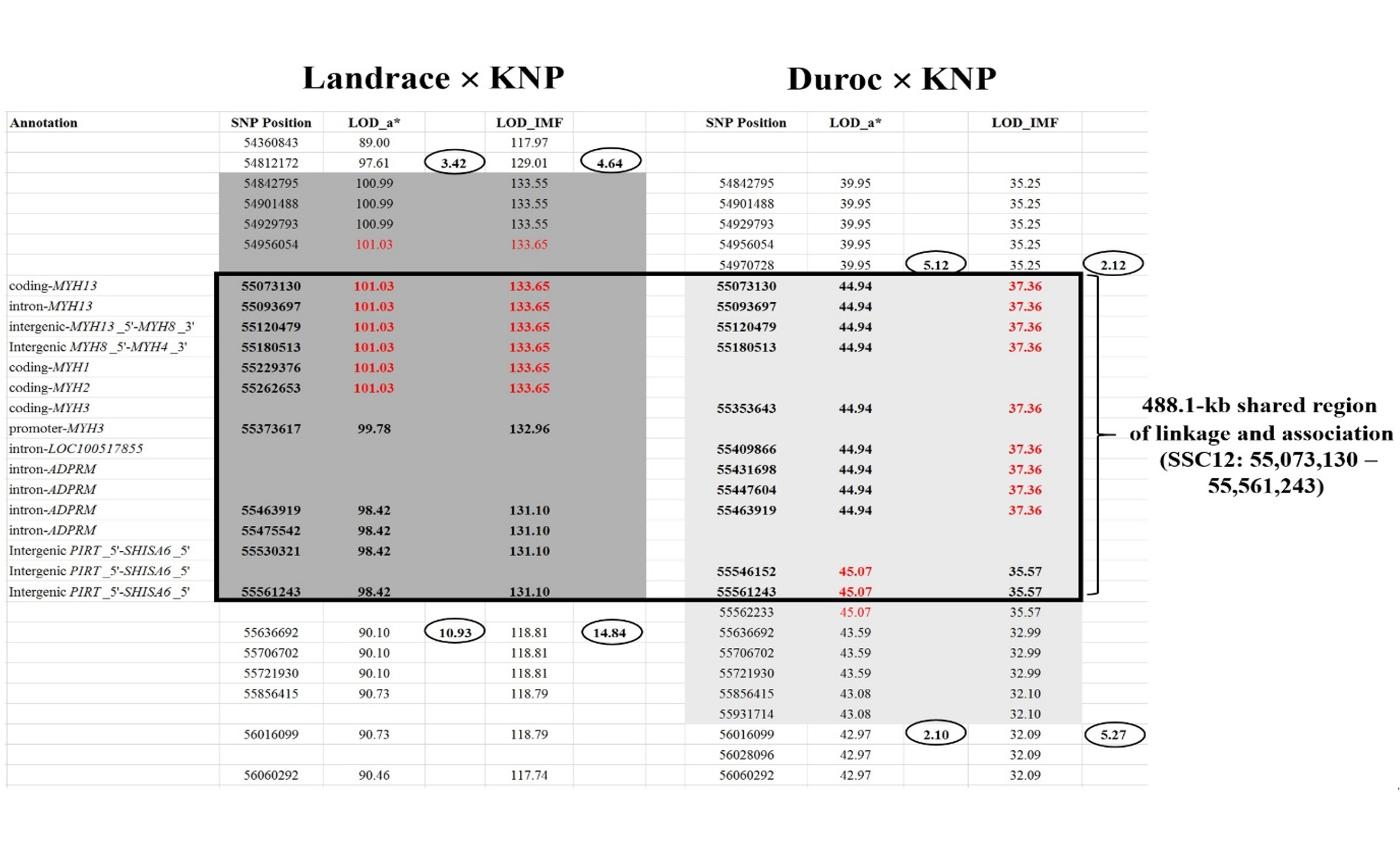

Supplement: S3 Fig — SNP position is the physical base pair position in SSC12 (Sus scrofa 11.1). LOD_a* and LOD_IMF represent the LOD (logarithm of odds) score for the redness meat color and intramuscular fat content. The red-colored values represent maximum LOD scores for a* and IMF traits in each intercross. The dark gray region represents the critical region (12:54,842,795–55,561,243) for the LK cross, while the light gray region (12:55,073,130–55,931,714) indicates the critical region for the DK cross. A conservative 2-LOD drop support interval was applied to estimate the critical region. The black box line is highlights the 488.1-kb shared critical region. Numbers in the ovals represent the LOD drop support. (TIF) [file pgen.1008279.s003.tif]

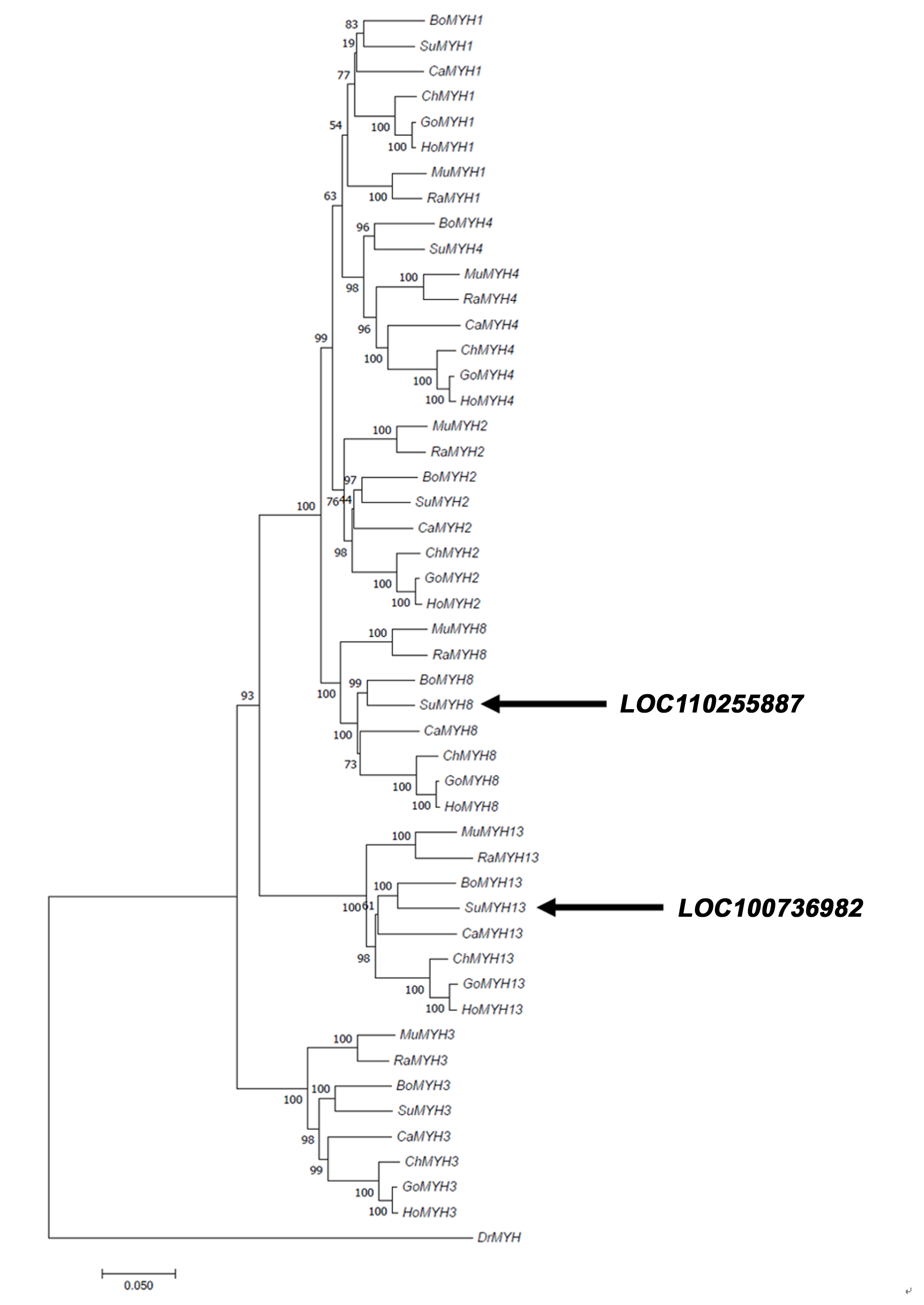

Supplement: S4 Fig — Multiple sequence alignment was performed with DIALIGN2.2.1 (URL:dialign.gobics.de). Numbers at the nodes represent the bootstrap support values derived from 10,000 replicates. The scale indicates the genetic distance. The accession numbers for the mRNA sequences are provided in Table S1. The species used are as follows: Bo (cattle), Ch (green monkey), Ca (dog), Go (Gorilla), Ho (human), Mu (mouse), Ra (rat), and Su (pig). We used the Dr (fruit fly) MYH mRNA sequence (NM_165190.4) as the out group. The MYH isoforms formed distinct clusters and this result provided conclusive evidence that MYH13 (LOC100736982) and MYH8 (LOC110255887) have been identified in pigs by this analysis. (TIF) [file pgen.1008279.s004.tif]

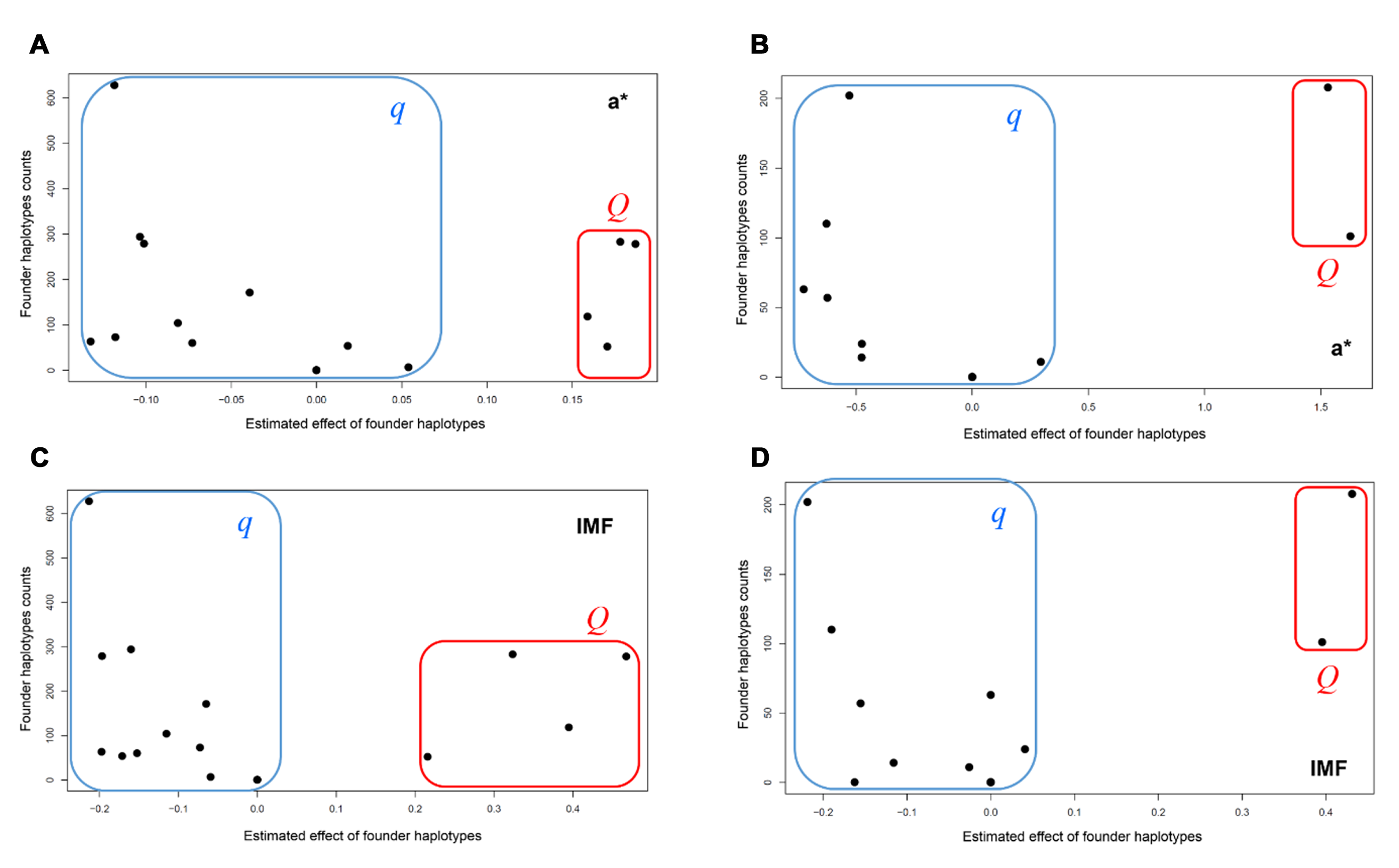

Supplement: S5 Fig — Founder haplotypes showing similar effects were pooled. Founder haplotypes associated with the inferred q and Q alleles of the later-detected as candidate functional sequence variants are shown in blue and red boxes, respectively. Phenotype data in the three panels (A, C and D) were natural log transformed. (A, C) For LK cross; (B, D) For DK cross. (TIF) [file pgen.1008279.s005.tif]

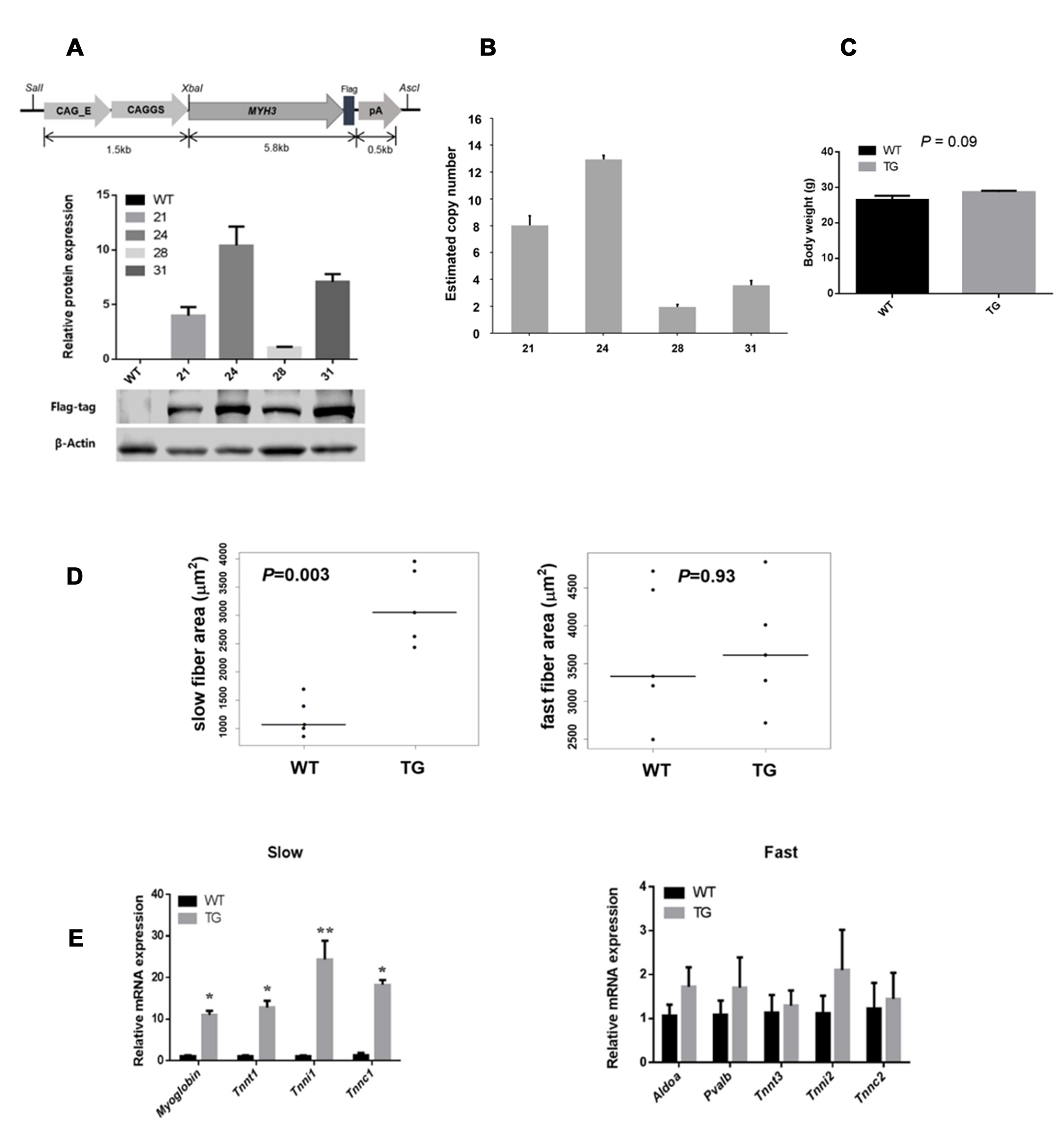

Supplement: S6 Fig — (A) Transgenic construction of the porcine MYH3 vector. The construct consists of the CAG promoter, porcine MYH3 mRNA sequence, flag for protein detection and pA (poly A) (upper panel). Western blotting analysis revealed that the 24 F1 founder showed the highest expression of MYH3 protein. The x-axis represents TG-mouse id. (B) Estimated porcine MYH3 transgene copy number in each TG. The x-axis represents TG-mouse id. The porcine MYH3 copy number ranged from 2 to 13 in each TG-mouse. (C) Body weight comparison between WT (n = 3) and TG (n = 4) mice. Body weights of male mice were measured at 4 months of age. (D) Comparison of the area of slow (type1/oxidative) and fast (type2) muscle fibers between WT (n = 5) and TG (n = 5) mice. The horizontal bars indicate median. (E) Expression of slow and fast muscle-associated genes in quadriceps muscle. Analyses of slow-type (left) and fast-type (right) muscle- associated gene expression by qRT-PCR. Four-month-old WT (n = 3) and TG (n = 4) mice were used. Data are mean±standard error for three independent replicates. *P<0.05, **P<0.01. (TIF) [file pgen.1008279.s006.tif]

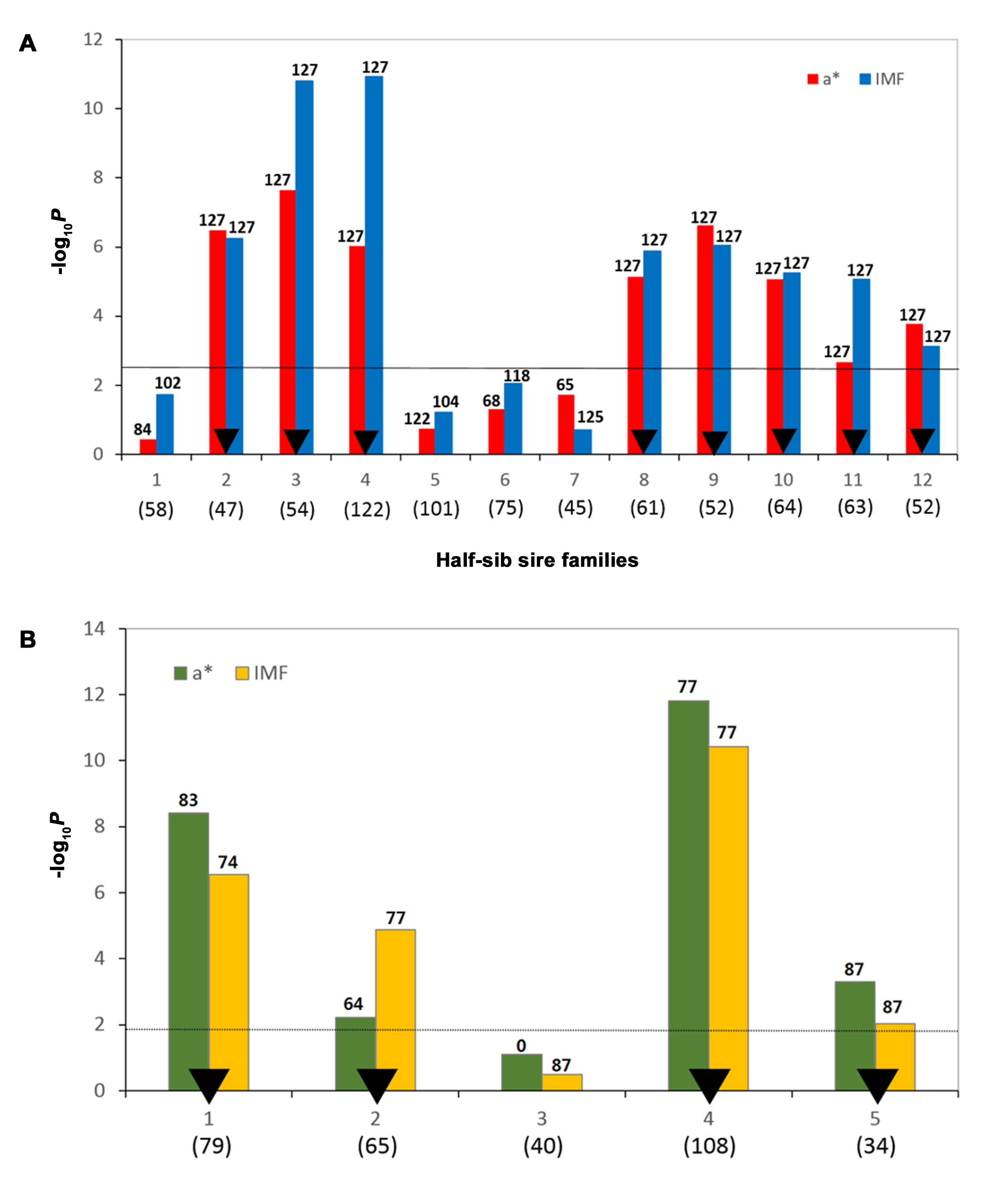

Supplement: S7 Fig — (A) Twelve for the LK cross; (B) Five for the DK cross. The chromosome-wide significance levels (1% for A; 5% for B) obtained from 10,000 permutations are shown as horizontal lines. Numbers above the bar graph correspond to the most likely chromosome position of QTL (cM). The numbers in the parentheses represent the number of progeny in each sire family. The black triangles indicate the sire families segregating for QTL. (TIF) [file pgen.1008279.s007.tif]

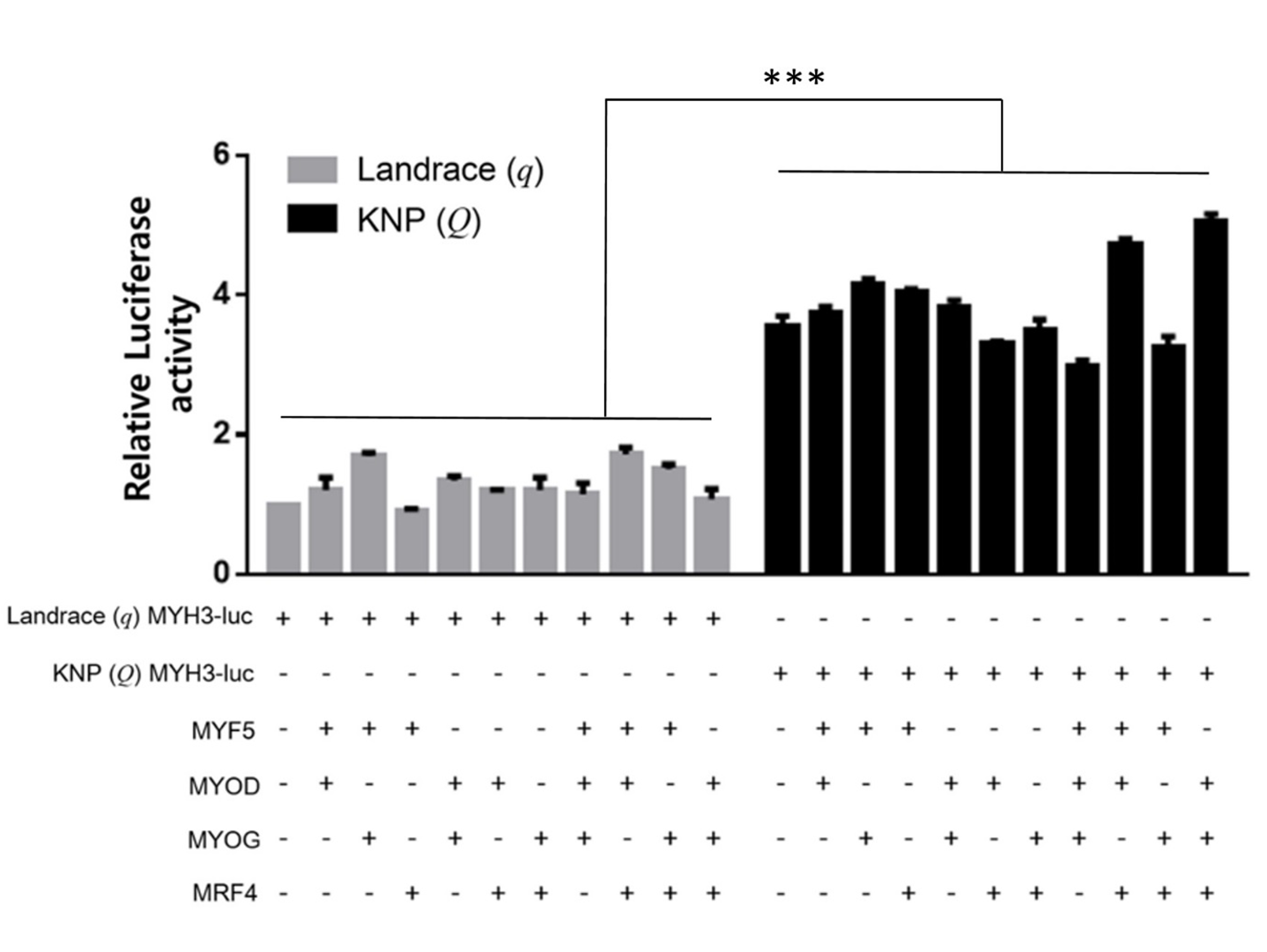

Supplement: S8 Fig — Reporter and MRFs constructs were electroporated in porcine fibroblast cells (MYH3 Luc, ‘empty’ vector cotransfected with MRF constructs). Luciferase activity of KNP (Q) was compared with that of Landrace (q). The MYH3 KNP (Q) promoter acts as a weaker repressor than the MYH3 Landrace (q) promoter. Data histograms and error bars represent the mean±standard error of three independent samples. ***P<0.001. (TIF) [file pgen.1008279.s008.tif]

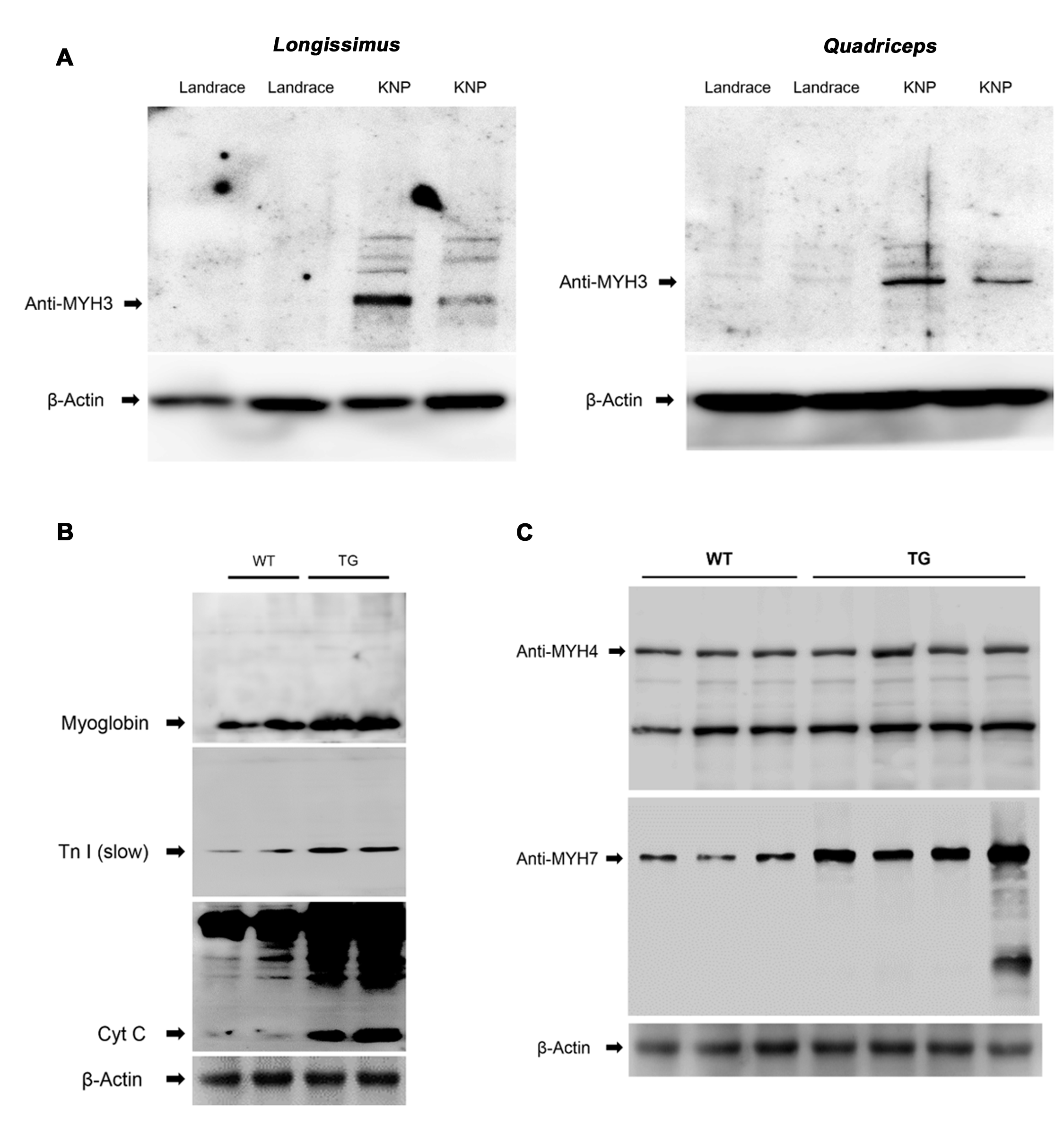

Supplement: S9 Fig — (A) Full blot figures of Fig 2E; (B) Full blot figures of Fig 3C; (C) Full blot figures of Fig 3D. (TIF) [file pgen.1008279.s009.tif]

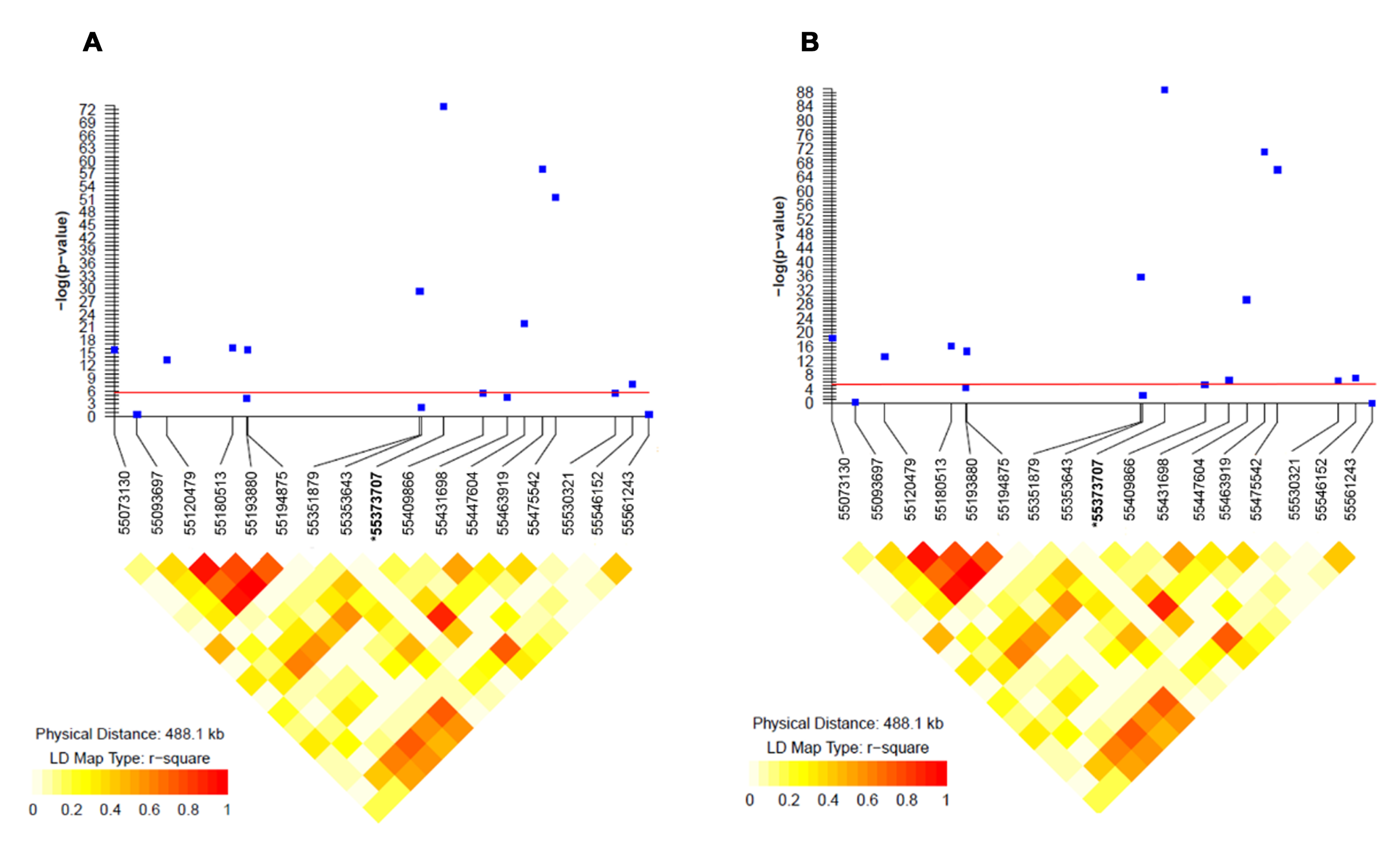

Supplement: S10 Fig — The y-axis shows the −log10(P-value), and the x-axis shows the physical positions of the SNP markers on the pig chromosome 12. The genome-wide significance threshold value is 5.90, which equals Bonferroni’s correction of 5% (represented by the red horizontal lines). There are lines to connect the pairwise LD structure with a black horizontal line representing the 488.1-kb critical region. The physical position of each SNP marker is demonstrated above the LD plot. The * indicates the position of MYH3 FSV. The magnitude of LD by r-square statistic is shown. (A) For a* in the LK cross (n = 963); (B) For IMF in the LK cross (n = 962). (TIF) [file pgen.1008279.s010.tif]
