# Supplementary material for: A functional regulatory variant of MYH3 influences muscle fiber-type composition and intramuscular fat content in pigs
Source: PLoS Genet. 2019 Oct 11;15(10):e1008279. doi: 10.1371/journal.pgen.1008279 (PMC6788688; doi:10.1371/journal.pgen.1008279)
Supplement: S1 Table — (DOCX) [file pgen.1008279.s011.docx]

**S1 Table.** Messenger RNA sequence identification, Refseq name, and physical position used for the phylogenetic analysis

| Species/Gene | *MYH1* | | *MYH2* | *MYH3* | *MYH4* | *MYH8* | *MYH13* |
| --- | --- | --- | --- | --- | --- | --- | --- |
| Cattle  (*Bos taurus, Bo*) | | NM_174117.1  AC_000176.1  (19:30110728-30135083) | NM_001166227.1  AC_000176.1  (19:30137767-30165109) | NM_001101835.1  AC_000176.1  (19:30230160-30251091) | *XM_002695806.4  AC_000176.1  (19:30080589-30103457) | NM_001206174.2  AC_000176.1  (19:30031027-30061053) | *XM_015458752.1  AC_000176.1  (19:29958329-30001291) |
| Green monkey  (*Chlorocebus sabaeus, Ch*) | | *XM_008010369.1  NC_023657.1  (16:9834434-9860744) | *XM_008010367.1  NC_023657.1  (16:9863336-9891271) | *XM_008010373.1  NC_023657.1  (16:9966801-9999458) | *XM_008010370.1  NC_023657.1  (16:9786143-9811908) | *XM_008010372.1  NC_023657.1  (16:9733585-9765740) | *XM_008010363.1  NC_023657.1  (16:9647080-9726679) |
| Dog  (*Canis lupus familiaris, Ca*) | | NM_001113717.1  NC_006587.3  (5:34781364-34808265) | NM_001076795.1  NC_006587.3  (5:34810793-34838177) | NM_001113712.1  NC_006587.3  (5:34914797-34938263) | NM_001076794.1  NC_006587.3  (5:34743454-34767076) | NM_001077235.1  NC_006587.3  (5:34696377-34725515) | *XM_003639035.2  NC_006587.3  (5:34617987-34669349) |
| Gorilla  (*Gorilla gorilla, Go*) | | *XM_019013360.1  NC_018441.2  (17:10561740-10587992) | *XM_019013357.1  NC_018441.2  (17:10590597-10619199) | *XM_019013359.1  NC_018441.2  (17:10703744-10734448) | *XM_004058586.2  NC_018441.2  (17:10513008-10538977) | *XM_019013364.1  NC_018441.2  (17:10460041-10491708) | *XM_019013356.1  NC_018441.2  (17:10370490-10442658) |
| Human  (*Homo sapiens, Ho*) | | NM_005963.3  NC_000017.11  (17:10492307-10518719) | *NM_001100112.1  NC_000017.11  (17:10521148-10549700) | NM_002470.3  NC_000017.11  (17:10628526-10679657) | NM_017533.2  NC_000017.11  (17:10443058-10469559) | NM_002472.2  NC_000017.11  (17:10390325-10421950) | NM_003802.2  NC_000017.11  (17:10300866-10373005) |
| Mouse  (*Mus musculus*, *Mu*) | | NM_030679.1  NC_000077.6  (11:67200109-67224575) | NM_001039545.2  NC_000077.6  (11:67171027-67197517) | NM_001099635.1  NC_000077.6  (11:67078273-67102291) | NM_010855.3  NC_000077.6  (11:67237812-67260447) | NM_177369.3  NC_000077.6  (11:67277124-67308634) | NM_001081250.1  NC_000077.6  (11:67327103-67371502) |
| Rat  (*Rattus norvegicus, Ra*) | | NM_001135158.1  NC_005109.4  (10:53740841-53764610) | NM_001135157.1  NC_005109.4  (10:53711895-53738164) | NM_012604.1  NC_005109.4  (10:53621375-53645194) | NM_019325.1  NC_005109.4  (10:53778456-53801605) | NM_001100485.1  NC_005109.4  (10:53818818-53848490) | *XM_017597793.1  NC_005109.4  (10:53873089-53920698) |
| Pig  (*Sus scrofa*, *Su*) | | NM_001104951.2  NC_010454.4  (12:55225581-55251495) | NM_214136.1  NC_010454.4  (12:55252974-55278820) | *XM_013981330.2  NC_010454.4  (12:55351152-55373236) | NM_001123141.1  NC_010454.4  (12:55190545-55216294) | *XM_021066269.1  NC_010454.4  (12:55135651-55166983) | *XM_021066284.1  NC_010454.4  (12:55051990-55103415) |

*Unannotated in NCBI database
