# Supplementary material for: A functional regulatory variant of MYH3 influences muscle fiber-type composition and intramuscular fat content in pigs
Source: PLoS Genet. 2019 Oct 11;15(10):e1008279. doi: 10.1371/journal.pgen.1008279 (PMC6788688; doi:10.1371/journal.pgen.1008279)
Supplement: S2 Table — (DOCX) [file pgen.1008279.s012.docx]

| **S2 Table.** Determination of QTL genotypes of F_1_ sires by marker assisted segregation analysis in LK and DK crosses (mean±standard error). | | | | | | | | | | | |
| --- | --- | --- | --- | --- | --- | --- | --- | --- | --- | --- | --- |
| Population | Animal ID |  |  | a* |  |  |  |  | IMF |  |  |
|  |  |  | Right  chromosome^1^ | Left chromosome^1^ | Log likelihood ratio | QTL genotype |  | Right chromosome^1^ | Left chromosome^1^ | Log  likelihood ratio | QTL genotype |
| LK F_1_ sires | 1 |  | 1.90±0.08 (19)^2^ | 1.84±0.06 (30) | −4.14 | Homo |  | 0.64±0.10 (19) | 0.78±0.07 (30) | −3.91 | Homo |
|  | 2 |  | 1.95±0.05 (23) | 2.26±0.04 (20) | 5.52 | **Hetero** |  | 0.59±0.06 (23) | 1.37±0.10 (20) | 6.78 | **Hetero** |
|  | 3 |  | 1.96±0.05 (17) | 2.46±0.03 (16) | 8.98 | **Hetero** |  | 0.51±0.07 (17) | 1.58±0.14 (16) | 6.05 | **Hetero** |
|  | 4 |  | 1.87±0.03 (49) | 2.26±0.04 (20) | 7.7 | **Hetero** |  | 0.58±0.04 (49) | 1.51±0.09 (21) | 11.07 | **Hetero** |
|  | 5 |  | 1.99±0.05 (41) | 2.00±0.06 (44) | −6.15 | Homo |  | 0.62±0.07 (41) | 0.74±0.07 (44) | −10.47 | Homo |
|  | 6 |  | 1.82±0.06 (21) | 1.84±0.04 (34) | −5.63 | Homo |  | 1.82±0.06 (21) | 1.84±0.04 (34) | −17.37 | Homo |
|  | 7 |  | 2.09±0.07 (20) | 2.15±0.08 (7) | −3.46 | Homo |  | 1.04±0.10 (20) | 1.24±0.13 (7) | −3.00 | Homo |
|  | 8 |  | 1.89±0.11 (14) | 2.53±0.06 (12) | 5.21 | **Hetero** |  | 0.63±0.09 (14) | 1.83±0.19 (10) | 6.58 | **Hetero** |
|  | 9 |  | 2.00±0.07 (18) | 2.44±0.08 (17) | 7.59 | **Hetero** |  | 0.66±0.08 (17) | 1.63±0.14 (17) | 8.99 | **Hetero** |
|  | 10 |  | 1.95±0.07 (18) | 2.37±0.06 (8) | 5.38 | **Hetero** |  | 0.67±0.13 (18) | 1.60±0.15 (8) | 3.81 | **Hetero** |
|  | 11 |  | 2.16±0.01 (8) | 2.63±0.04 (11) | 4.54 | **Hetero** |  | 0.65±0.09 (8) | 1.75±0.12 (11) | 4.32 | **Hetero** |
|  | 12 |  | 1.90±0.06 (16) | 2.33±0.03 (9) | 3.8 | **Hetero** |  | 0.50±0.07 (16) | 1.29±0.14 (9) | 9.34 | **Hetero** |
|  |  |  |  |  |  |  |  |  |  |  |  |
| DK F_1_ sires | 1 |  | 6.41±0.31 (15) | 10.16±0.14 (11) | 5.73 | **Hetero** |  | 0.94±0.11 (15) | 1.94±0.08 (11) | 5.07 | **Hetero** |
|  | 2 |  | 11.85±0.69 (13) | 2.11±0.11 (13) | 3.79 | **Hetero** |  | 2.11±0.11 (13) | 1.15±0.10 (12) | 5.23 | **Hetero** |
|  | 3 |  | 8.29±0.51 (8) | 7.72±1.1 (8) | −2.78 | Homo |  | 1.44±0.14 (8) | 1.09±0.21 (8) | −4.38 | Homo |
|  | 4 |  | 9.77±0.32 (24) | 6.66±0.18 (41) | 9.85 | **Hetero** |  | 2.08±0.10 (24) | 1.11±0.07 (41) | 9.4 | **Hetero** |
|  | 5 |  | 10.04±0.44 (5) | 6.82±0.17 (9) | 4.7 | **Hetero** |  | 1.81±0.34 (5) | 0.75±0.15 (9) | 2.54 | **Hetero** |
| ^1^Right and left chromosomes are arbitrarily assigned for each F_1_ sire.  ^2^The number in the parenthesis represents the numbers of F_2_ progeny in each group. | | | | | |  |  |  |  |  |  |
