# Supplementary material for: A functional regulatory variant of MYH3 influences muscle fiber-type composition and intramuscular fat content in pigs
Source: PLoS Genet. 2019 Oct 11;15(10):e1008279. doi: 10.1371/journal.pgen.1008279 (PMC6788688; doi:10.1371/journal.pgen.1008279)
Supplement: S3 Table — (DOCX) [file pgen.1008279.s013.docx]

**S3 Table.** Positions of overlapped putative FSVs located in predicted regulatory motifs in the 488.1-kb critical region

| No | BP^1^ | Candidate FSV | Annotation^2^ | Putative transcription factor^3^ |  |
| --- | --- | --- | --- | --- | --- |
| 1 | 55,075,726 | G>A | *intron-MYH13* | CHCH, ZBTB7A, AP2, SP1, CAC |  |
| 2 | 55,087,813 | G>T | *intron-MYH13* |  |  |
| 3 | 55,091,628 | A>T | *intron-MYH13* | FXR |  |
| 4 | 55,091,723 | C>T | *intron-MYH13* | MYF5 |  |
| 5 | 55,091,836 | C>T | *intron-MYH13* |  |  |
| 6 | 55,092,723 | A>C | *intron-MYH13* | SPZ1 |  |
| 7 | 55,095,793 | G>A | *intron-MYH13* |  |  |
| 8 | 55,100,558 | G>C | *intron-MYH13* |  |  |
| 9 | 55,100,562 | C>T | *intron-MYH13* |  |  |
| 10 | 55,103,461 | A>G | *promoter-MYH13* |  |  |
| 11 | 55,103,880 | C>T | *promoter-MYH13* |  |  |
| 12 | 55,106,596 | G>A | intergenic-*MYH13*_5'-*MYH8*_3' |  |  |
| 13 | 55,106,653 | G>A | intergenic-*MYH13*_5'-*MYH8*_3' |  |  |
| 14 | 55,106,705 | T>G | intergenic-*MYH13*_5'-*MYH8*_3' | AP2 |  |
| 15 | 55,107,054 | G>A | intergenic-*MYH13*_5'-*MYH8*_3' | AP2 |  |
| 16 | 55,107,607 | A>G | intergenic-*MYH13*_5'-*MYH8*_3' |  |  |
| 17 | 55,108,827 | G>C | intergenic-*MYH13*_5'-*MYH8*_3' |  |  |
| 18 | 55,110,634 | C>T | intergenic-*MYH13*_5'-*MYH8*_3' |  |  |
| 19 | 55,138,689 | G>T | *intron-MYH8* |  |  |
| 20 | 55,144,188 | C>T | *intron-MYH8* |  |  |
| 21 | 55,145,024 | G>A | *intron-MYH8* |  |  |
| 22 | 55,145,046 | C>T | *intron-MYH8* |  |  |
| 23 | 55,145,220 | G>A | *intron-MYH8* |  |  |
| 24 | 55,160,841 | C>T | *intron-MYH8* |  |  |
| 25 | 55,161,199 | C>T | *intron-MYH8* |  |  |
| 26 | 55,166,007 | C>T | *intron-MYH8* |  |  |
| 27 | 55,167,135 | T>C | *promoter-MYH8* |  |  |
| 28 | 55,170,389 | C>T | intergenic-*MYH8*_5'-*MYH4*_3' |  |  |
| 29 | 55,170,705 | G>A | intergenic-*MYH8*_5'-*MYH4*_3' |  |  |
| 30 | 55,171,928 | A>G | intergenic-*MYH8*_5'-*MYH4*_3' | br |  |
| 31 | 55,171,942 | G>A | intergenic-*MYH8*_5'-*MYH4*_3' |  |  |
| 32 | 55,171,954 | T>C | intergenic-*MYH8*_5'-*MYH4*_3' |  |  |
| 33 | 55,171,955 | G>A | intergenic-*MYH8*_5'-*MYH4*_3' |  |  |
| 34 | 55,189,362 | G>T | intergenic-*MYH8*_5'-*MYH4*_3' |  |  |
| 35 | 55,189,843 | G>A | intergenic-*MYH8*_5'-*MYH4*_3' |  |  |
| 36 | 55,190,041 | C>A | intergenic-*MYH8*_5'-*MYH4*_3' |  |  |
| 37 | 55,190,058 | T>C | intergenic-*MYH8*_5'-*MYH4*_3' |  |  |
| 38 | 55,209,830 | G>A | *intron-MYH4* |  |  |
| 39 | 55,209,964 | G>A | *intron-MYH4* |  |  |
| 40 | 55,212,550 | G>A | *intron-MYH4* |  |  |
| 41 | 55,212,835 | G>A | *intron-MYH4* |  |  |
| 42 | 55,217,726 | G>A | *promoter-MYH4* |  |  |
| 43 | 55,218,299 | G>A | intergenic-*MYH4*_5'-*MYH1*_3' |  |  |
| 44 | 55,219,196 | G>A | intergenic-*MYH4*_5'-*MYH1*_3' | RBPJ |  |
| 45 | 55,220,914 | A>G | intergenic-*MYH4*_5'-*MYH1*_3' | GATA |  |
| 46 | 55,222,475 | C>G | intergenic-*MYH4*_5'-*MYH1*_3' |  |  |
| 47 | 55,271,993 | C>T | *intron-MYH2* |  |  |
| 48 | 55,274,041 | A>T | *intron-MYH2* |  |  |
| 49 | 55,275,636 | C>T | *intron-MYH2* |  |  |
| 50 | 55,275,643 | T>G | *intron-MYH2* |  |  |
| 51 | 55,275,885 | G>A | *intron-MYH2* |  |  |
| 52 | 55,275,888 | C>A | *intron-MYH2* |  |  |
| 53 | 55,276,153 | A>G | *intron-MYH2* |  |  |
| 54 | 55,277,062 | A>G | *intron-MYH2* |  |  |
| 55 | 55,277,534 | G>C | *intron-MYH2* | GATA |  |
| 56 | 55,277,826 | C>T | *intron-MYH2* |  |  |
| 57 | 55,277,904 | C>T | *intron-MYH2* |  |  |
| 58 | 55,279,234 | C>T | *promoter-MYH2* | GATA |  |
| 59 | 55,280,351 | A>G | *promoter-MYH2* |  |  |
| 60 | 55,287,735 | G>A | intergenic-*MYH2*_5'-*MYH3*_3' | STAT |  |
| 61 | 55,288,930 | G>A | intergenic-*MYH2*_5'-*MYH3*_3' |  |  |
| 62 | 55,293,800 | C>T | intergenic-*MYH2*_5'-*MYH3*_3' | br, EVI1, STAT, PAX |  |
| 63 | 55,294,903 | G>T | intergenic-*MYH2*_5'-*MYH3*_3' |  |  |
| 64 | 55,303,500 | C>T | intergenic-*MYH2*_5'-*MYH3*_3' |  |  |
| 65 | 55,305,979 | C>G | intergenic-*MYH2*_5'-*MYH3*_3' | SP1 |  |
| 66 | 55,310,437 | G>T | intergenic-*MYH2*_5'-*MYH3*_3' | CDX |  |
| 67 | 55,311,529 | C>A | intergenic-*MYH2*_5'-*MYH3*_3' |  |  |
| 68 | 55,314,369 | A>G | intergenic-*MYH2*_5'-*MYH3*_3' |  |  |
| 69 | 55,314,583 | A>G | intergenic-*MYH2*_5'-*MYH3*_3' | MYOG |  |
| 70 | 55,334,734 | A>G | intergenic-*MYH2*_5'-*MYH3*_3' | SP1, CHCH |  |
| 71 | 55,341,263 | G>A | intergenic-*MYH2*_5'-*MYH3*_3' | HIC1, ELK |  |
| 72 | 55,341,904 | G>A | intergenic-*MYH2*_5'-*MYH3*_3' |  |  |
| 73 | 55,357,336 | A>T | *intron-MYH3* |  |  |
| 74 | 55,357,355 | G>A | *intron-MYH3* |  |  |
| 75 | 55,357,598 | G>A | *intron-MYH3* |  |  |
| 76 | 55,372,877 | T>C | *intron-MYH3* | SREBP |  |
| 77 | 55,373,707 | CAGTTC>**−** | *promoter-MYH3* | ^*^MYF5, MYOD, MYOG, MRF4 |  |
| 78 | 55,375,854 | C>A | intergenic-*MYH3*_5'-*LOC100517855*_3' |  |  |
| 79 | 55,378,550 | A>G | intergenic-*MYH3*_5'-*LOC100517855*_3' |  |  |
| 80 | 55,449,550 | A>G | *intron-TMEM220* |  |  |
| 81 | 55,461,962 | A>G | intergenic-*TEME220*_5'-*LOC110255888*_3' |  |  |
| 82 | 55,475,293 | C>T | intergenic-*TEME220*_5'-*LOC110255888*_3' |  |  |
| 83 | 55,496,565 | G>C | intergenic-*TEME220*_5'-*LOC110255888*_3' |  |  |
| 84 | 55,501,010 | G>A | intergenic-*LOC110255888*_5'-*PIRT*_3' |  |  |
| 85 | 55,503,703 | T>C | intergenic-*LOC110255888*_5'-*PIRT*_3' |  |  |
| 86 | 55,503,849 | T>C | intergenic-*LOC110255888*_5'-*PIRT*_3' |  |  |
| 87 | 55,503,978 | T>A | intergenic-*LOC110255888*_5'-*PIRT*_3' | NFE2 |  |
| 88 | 55,504,586 | C>T | intergenic-*LOC110255888*_5'-*PIRT*_3' | SREBP |  |
| 89 | 55,505,247 | A>T | intergenic-*LOC110255888* 5'-*PIRT*_3' |  |  |
| 90 | 55,505,264 | C>G | intergenic-*LOC110255888* 5'-*PIRT*_3' |  |  |
| ^1^Physical position of putative FSV located in putative regulatory motifs predicted by MEME in the 488.1-kb critical region.  ^2^intron (an intron region); promoter (within 2-kb of a transcription initiation site); intergenic (all other variants between genes). Each candidate FSV was assigned to  a unique annotation defined by one of the three categories. | | | | | |
| ^3^Putative transcription factor binding sites predicted by TRAP.  ^*^Putative transcription factor binding sites predicted by TRAP, JASPAR, and PROMO. | | | |  |  |
