# Supplementary material for: A functional regulatory variant of MYH3 influences muscle fiber-type composition and intramuscular fat content in pigs
Source: PLoS Genet. 2019 Oct 11;15(10):e1008279. doi: 10.1371/journal.pgen.1008279 (PMC6788688; doi:10.1371/journal.pgen.1008279)
Supplement: S4 Table — (DOCX) [file pgen.1008279.s014.docx]

| **S4 Table** Results of CAVIAR and eCAVIAR analyses using the Porcine60K BeadChip chip variants in the 488.1-kb critical region | | | | | | | | | |
| --- | --- | --- | --- | --- | --- | --- | --- | --- | --- |
| No | ^1^BP | Variant annotation | ^2^Freq | | ^3^Probability a* | | ^4^Probability IMF | ^5^CLPP a* and IMF | |
| 1 | 55,073,130 | coding-*MYH13* | | 0.36 | | 1.17×10^−55^ | 1.27×10^−71^ | 1.48×10^−126^ | |
| 2 | 55,093,697 | intron*-MYH13* | | 0.21 | | 2.85×10^−67^ | 1.01×10^−85^ | 2.86×10^−152^ | |
| 3 | 55,120,479 | intergenic-*MYH13_5′-MYH8_3′* | | 0.42 | | 1.27×10^−57^ | 7.22×10^−76^ | 9.16×10^−133^ | |
| 4 | 55,180,513 | intergenic-*MYH8_5′-MYH4_3′* | | 0.43 | | 2.81×10^−55^ | 1.81×10^−73^ | 5.09×10^−128^ | |
| 5 | 55,193,880 | intron*-MYH4* | | 0.46 | | 1.10×10^−64^ | 6.90×10^−83^ | 7.61×10^−147^ | |
| 6 | 55,194,875 | exon*-MYH4* | | 0.43 | | 1.13×10^−55^ | 1.06×10^−74^ | 1.19×10^−129^ | |
| 7 | 55,351,879 | intron-*MYH3* | | 0.46 | | 1.00×10^−43^ | 2.76×10^−56^ | 2.76×10^−99^ | |
| 8 | 55,353,643 | exon-*MYH3* | | 0.17 | | 3.36×10^−66^ | 1.58×10^−84^ | 5.30×10^−150^ | |
| **9** | **55,373,707** | **promoter-*MYH3*** | | **0.36** | | **1.00^§^** | **1.00^§^** | **1.00^§^** | |
| 10 | 55,409,866 | intron-*LOC100517855* | | 0.31 | | 7.67×10^−64^ | 3.30×10^−82^ | 2.53×10^−145^ | |
| 11 | 55,431,698 | intron-*ADPRM* | | 0.29 | | 2.02×10^−64^ | 2.81×10^−81^ | 5.68×10^−145^ | |
| 12 | 55,447,604 | intron-*ADPRM* | | 0.21 | | 2.23×10^−50^ | 3.47×10^−62^ | 7.73×10^−112^ | |
| 13 | 55,463,919 | intron-*ADPRM* | | 0.38 | | 3.19×10^−16^ | 1.06×10^−20^ | 3.39×10^−36^ | |
| 14 | 55,475,542 | intergenic-*TMEM220_5′-LOC110255888_3′* | | 0.43 | | 4.03×10^−23^ | 3.58×10^−26^ | 1.44×10^−48^ | |
| 15 | 55,530,321 | intergenic-*PIRT_5′-SHISA_5′* | | 0.07 | | 1.03×10^−63^ | 2.08×10^−81^ | 2.14×10^−114^ | |
| 16 | 55,546,152 | intergenic-*PIRT_5′-SHISA_5′* | | 0.36 | | 3.87×10^−62^ | 1.00×10^−80^ | 3.87×10^−142^ | |
| 17 | 55,561,243 | intergenic-*PIRT_5′-SHISA_5′* | | 0.36 | | 2.61×10^−67^ | 9.02×10^−86^ | 2.36×10^−152^ | |
| ^1^BP: physical position of variants in the 488.1-kb critical region on SSC12 from the Porcine60K BeadChip and *MYH3−1805_−1810delCAGTCC*. | | | | | | | | | |
| ^2^Freq: minor allele frequency of each variant in the LK cross. | | | | |  | |  |  |  |
| ^3^Probability a*: posterior probability of being a candidate causal variant for a* computed using CAVIAR. | | | | | | | | |  |
| ^4^Probability IMF: posterior probability of being a candidate causal variant for IMF computed using CAVIAR. | | | | | | | | |  |
| ^5^CLPP a* and IMF: combined likelihood posterior probability of being a candidate causal variant for both a* and IMF computed using eCAVIAR. | | | | | | | | | |
| ^§^Variants included in the 99% credible set of being causal.  Bold characters represent *MYH3−1805_−1810delCAGTCC* and its results. | | | | |  | |  |  |  |
