# Supplementary material for: A functional regulatory variant of MYH3 influences muscle fiber-type composition and intramuscular fat content in pigs
Source: PLoS Genet. 2019 Oct 11;15(10):e1008279. doi: 10.1371/journal.pgen.1008279 (PMC6788688; doi:10.1371/journal.pgen.1008279)
Supplement: S5 Table — (DOCX) [file pgen.1008279.s015.docx]

**S5 Table.** Allele frequency of the *MYH3* FSV among pig populations

| Breed |  | n | *p*(*Q*) |
| --- | --- | --- | --- |
| European wild boar | Polish wild boar | 4 | 0.00 |
|  | Romanian wild boar | 4 | 0.00 |
|  | Swedish wild boar | 3 | 0.00 |
|  | Slovenian wild boar | 4 | 0.00 |
|  | Spanish wild boar | 1 | 0.00 |
| European domestic pig | Large White | 30 | 0.00 |
|  | Landrace | 17 | 0.00 |
|  | Berkshire | 30 | 0.02 |
|  | Hampshire | 2 | 0.00 |
|  | Duroc | 30 | 0.00 |
|  | Iberian | 6 | 0.00 |
|  | United Kingdom Middle White | 1 | 1.00 |
|  | United Kingdom Large Black | 2 | 0.00 |
|  | United Kingdom Saddle Back | 2 | 0.00 |
|  | United Kingdom Old Spot | 1 | 0.00 |
|  | United Kingdom British Lop | 1 | 0.00 |
|  | United Kingdom Chester White | 4 | 0.00 |
| African wild boar | Tunisian wild boar | 6 | 0.00 |
|  | Moroccan wild boar | 1 | 0.00 |
| Asian wild boar | Korean wild boar | 90 | 0.13 |
|  | Russian wild boar (Primosrky Krai) | 9 | 0.22 |
| Asian domestic pig | Korean native (Jeju) | 51 | 0.63 |
|  | Chinese Jinhua | 6 | 0.08 |
|  | Chinese Min | 7 | 0.21 |
|  | Chinese Neijang | 5 | 0.80 |
|  | Chinese Putian | 5 | 0.80 |
|  | Chinese Tongcheng | 10 | 0.90 |
|  | Chinese Wannanhua | 5 | 0.00 |
|  | Chinese Wuzhishan | 17 | 0.00 |
|  | Chinese Xiang | 23 | 0.54 |

n, number of pigs; *p*(*Q*) = allele frequency of the *MYH3* FSV associated with increased a* and IMF.
