# Supplementary material for: A functional regulatory variant of MYH3 influences muscle fiber-type composition and intramuscular fat content in pigs
Source: PLoS Genet. 2019 Oct 11;15(10):e1008279. doi: 10.1371/journal.pgen.1008279 (PMC6788688; doi:10.1371/journal.pgen.1008279)
Supplement: S6 Table — (DOCX) [file pgen.1008279.s016.docx]

S6 Table. Nucleotide diversities per base pair and Tajima's D statistics by region and by pig population, obtained from re-sequencing data.

| Population/Breed | n | Region (SSC12, assembly 11.1 coordinates) | | | | | |
| --- | --- | --- | --- | --- | --- | --- | --- |
|  |  | Critical (55,073,130 -55,561,243) | | *MYH3* (55,351,152 -55,373,236) | | Promoter (55,373,237-55,375,237) | |
|  |  | π | Tajima's D | π | Tajima's D | π | Tajima's D |
| Korean wild | 10 | 0.0059 | 1.50 | 0.0048 | 0.71 | 0.0055 | 0.75 |
| Asian domestic^a^ | 24 | 0.0059 | 1.25 | 0.0054 | 0.42 | 0.0048 | 1.48 |
| Korean Native Pig | 10 | 0.0047 | 2.31 | 0.0039 | 0.40 | 0.0040 | 2.50 |
| Meishan | 10 | 0.0060 | 1.03 | 0.0058 | 0.36 | 0.0064 | 1.75 |
| Tongcheng | 4 | 0.0070 | 0.87 | 0.0054 | 0.39 | 0.0082 | 0.32 |
| European wild^b^ | 10 | 0.0019 | −0.10 | 0.0041 | 0.50 | 0.0015 | −1.56 |
| European domestic^c^ | 41 | 0.0038 | 0.78 | 0.0055 | 1.63 | 0.0069 | 0.34 |
| Iberian | 6 | 0.0024 | 1.29 | 0.0059 | 1.77 | 0.0071 | 0.00 |
| Large White | 10 | 0.0035 | 0.13 | 0.0048 | 0.79 | 0.0074 | 0.57 |
| Landrace | 10 | 0.0050 | 1.82 | 0.0065 | 1.99 | 0.0067 | 1.77 |
| Duroc | 10 | 0.0010 | 2.05 | 0.0019 | 2.05 | 0.0036 | 1.86 |
| Berkshire | 5 | 0.0015 | 1.23 | 0.0057 | 1.59 | 0.0036 | −0.61 |

^a^ Comprises all KNP, Meishan and Toncheng pigs together.

^b^ Comprises boars from Spain, France, Holland, Switzerland, Italy and Tunis.

^c^ Comprises all Iberian, Large White, Landrace, Duroc and Berkshire pigs.
