# Supplementary material for: A functional regulatory variant of MYH3 influences muscle fiber-type composition and intramuscular fat content in pigs
Source: PLoS Genet. 2019 Oct 11;15(10):e1008279. doi: 10.1371/journal.pgen.1008279 (PMC6788688; doi:10.1371/journal.pgen.1008279)
Supplement: S7 Table — (DOCX) [file pgen.1008279.s017.docx]

S7 Table. qRT-PCR primers for analysis of mouse muscle samples

| Accession Number | Gene | Forward | Reverse | Product size (bp) |
| --- | --- | --- | --- | --- |
| NM_001361607.1 | *Myh7* | AGTCCCAGGTCAACAAGCTG | TTCCACCTAAAGGGCTGTTG | 146 |
| NM_001039545.2 | *Myh2* | AGTCCCAGGTCAACAAGCTG | GCATGACCAAAGGTTTCACA | 130 |
| NM_030679.2 | *Myh1* | AGTCCCAGGTCAACAAGCTG | CACATTTTGCTCATCTCTTTG | 113 |
| NM_010855.3 | *Myh4* | AGTCCCAGGTCAACAAGCTG | TTTCTCCTGTCACCTCTCAACA | 100 |
| NM_001164047.1 | *Myoglobin* | GCAAGGCCCTGGAGCTCT | GCTTGGTGGGCTGGACAGTG | 100 |
| NM_001277903.1 | *Tnnt1* | CCCCCGAAGATTCCAGAAGG | TGCGGTCTTTTAGTGCAATGAG | 154 |
| NM_001112702.1 | *Tnni1* | ATGCCGGAAGTTGAGAGGAAA | TCCGAGAGGTAACGCACCTT | 140 |
| NM_009393.3 | *Tnnc1* | GCGGTAGAACAGTTGACAGAG | CCAGCTCCTTGGTGCTGAT | 103 |
| NM_001177307.1 | *Aldoa* | ACTCTCTGCTGACCGGGCTCT | AATGCTTCCGGTGGACTCAT | 186 |
| NM_001330686.1 | *Pvalb* | ATCAAGAAGGCGATAGGAGCC | GGCCAGAAGCGTCTTTGTT | 231 |
| NM_001163664.1 | *Tnnt3* | GGAACGCCAGAACAGATTGG | TGGAGGACAGAGCCTTTTTCTT | 104 |
| NM_009405.3 | *Tnni2* | AGAGTGTGATGCTCCAGATAGC | AGCAACGTCGATCTTCGCA | 170 |
| NM_009394.2 | *Tnnc2* | ATGGCAGCGGTACTATCGACT | CCTTCGCATCCTCTTTCATCTG | 72 |
| NM_001159555.1 | *CD36* | AATGGCACAGACGCAGCCT | GGTTGTCTGGATTCTGGA | 190 |
| NM_008509.2 | *LPL* | GTACCTGAAGACTCGCTCTC | AGGGTGAAGGGAATGTTCTC | 156 |
| NM_024406.3 | *Fabp4* | GATGCCTTTGTGGGAACCTG | TCCTGTCGTCTGCGGTGATT | 232 |
| NM_011936.2 | *Fto* | GTCAGAGAGAAGGCCAATGA | TAGCAGTCTCCCTGGTGAAG | 401 |
| NM_008904.2 | *Pgc1α* | CCCTGCCATTGTTAAGACC | TGCTGCTGTTCCTGTTTTC | 161 |
| NM_009605.5 | *Adiponectin* | AATGGCACACCAGGCCGTGAT | TCTCCAGGCTCTCCTTTCCTG | 170 |
| NM_001127330.2 | *GAPDH* | GAAACTCTGGGAGATTCTCCT | CAGAGCTGATTCCGAAGTTGG | 136 |
