# Supplementary material for: A functional regulatory variant of MYH3 influences muscle fiber-type composition and intramuscular fat content in pigs
Source: PLoS Genet. 2019 Oct 11;15(10):e1008279. doi: 10.1371/journal.pgen.1008279 (PMC6788688; doi:10.1371/journal.pgen.1008279)
Supplement: S8 Table — (DOCX) [file pgen.1008279.s018.docx]

| Accession Number | Gene | Forward | Reverse | Product size (bp) |
| --- | --- | --- | --- | --- |
| XM_021066284.1 | *^1^LOC100736982* | CCTACAAGTGGCTGCCGGTG | GTTCACCGTCTTCCCAGCCC | 182 |
| XM_021066269.1 | *^2^LOC110255887* | GAAGCCGACAGCGGCACAAA | AGATGCGGATGCCCTCCA | 232 |
| XM_021066035.1 | *pMYH4* | TCTGAAGAGGGTGGTACAAAGA | AGATGCGGATGCCCTCCA | 236 |
| NM_001104951.2 | *pMYH1* | TGATGCAGAGGCTGGAGGTG | AGATGCGGATGCCCTCCA | 236 |
| NM_214136.1 | *pMYH2* | GGGCTCAAACTGGTGAAGC | AGATGCGGATGCCCTCCA | 249 |
| XM_013981330.2 | *pMYH3* | GCCGACGCTGACAGCGGAAA | AGATGCGGATGCCCTCCA | 235 |
| XM_003131996.4 | *LOC100517855* | TCCTCACGGACTCGGGGTTT | GTGGGGTCTCTGCTGCCCTT | 198 |
| XM_003131997.5 | *pTMEM220* | CCCAGACGCAGAACTGTGGG | GTTGTATGCCAAGCCGGCAG | 160 |
| XM_021066275.1 | *pADPRM* | CATCCTGAGACCGTGCCTTCA | TTCCGCATTTGGGTTGTGCT | 174 |
| XM_021066278.1 | *LOC110255888* | CCACGTTCCTGATTCACGGG | TTTGGAGGACGAGCTGGGAA | 130 |
| XM_021066282.1 | *pPIRT* | GAATCCAAGGACCTGCTGCC | CCCACAGACATGATGACGGC | 131 |
| NM_001206359 1 | *pGAPDH* | GGGCATGAACCATGAGAAGT | AAGCAGGGATGATGTTCTGG | 162 |

**S8 Table.** qRT-PCR primers for analysis of muscle samples from pigs
