# Supplementary material for: A functional regulatory variant of MYH3 influences muscle fiber-type composition and intramuscular fat content in pigs
Source: PLoS Genet. 2019 Oct 11;15(10):e1008279. doi: 10.1371/journal.pgen.1008279 (PMC6788688; doi:10.1371/journal.pgen.1008279)
Supplement: S9 Table — (DOCX) [file pgen.1008279.s019.docx]

**S9 Table.** List of antibodies used in this study

| **First antibody** | **Number** | **Source** | **Application^*^** |
| --- | --- | --- | --- |
| Anti-Flag M2 | F1804 | Sigma-Aldrich | WB, IHC |
| Anti-HA | H6908 | Sigma-Aldrich | WB |
| MYH4 | H00004622-B01P | Abnova | WB |
| MYH4 | 20140-1-AP | Proteintech | ICH |
| MYH7 | SC-53089 | Santa cruz biotechnology | WB, IHC |
| MYH3 | Orb385438 | Biorbyt | WB |
| Myoglobin | SC-8081 | Santa cruz biotechnology | WB |
| Troponin I-SS | SC-8119 | Santa cruz biotechnology | WB |
| Cytochrom C | SC-13156 | Santa cruz biotechnology | WB |
| β-actin | #4970 | Cell signaling | WB |
| **Secondary antibody** | **Number** | **Source** | **Application^*^** |
| Donkey anti-Rabbit IgG (H+L) | 31821 | Thermo | WB |
| Mouse anti-goat IgG | sc-2489 | Santa cruz biotechnology | WB |
| Goat anti-mouse IgG | sc-2005 | Santa cruz biotechnology | WB |
| Chicken anti-Rabbit IgG (H+L) Cross-Adsorbed, Alexa Fluor® 488 | A21441 | Thermo | IHC |
| Goat anti-Mouse IgG (H+L) Cross-Adsorbed, Alexa Fluor® 488 | A11001 | Thermo | IHC |
| Goat anti-Mouse IgG (H+L) Cross-Adsorbed, Alexa Fluor® 555 | A21422 | Thermo | IHC |

*WB: Western blotting; IHC: immunohistochemistry
