# Supplementary material for: A functional regulatory variant of MYH3 influences muscle fiber-type composition and intramuscular fat content in pigs
Source: PLoS Genet. 2019 Oct 11;15(10):e1008279. doi: 10.1371/journal.pgen.1008279 (PMC6788688; doi:10.1371/journal.pgen.1008279)
Supplement: S10 Table — (DOCX) [file pgen.1008279.s020.docx]

**S10 Table. Resequencing data access information**

| **id** | **Breed** | **Country** | **Sex** | **Genbank Accession** | **SRA run accession** |
| --- | --- | --- | --- | --- | --- |
| WBKR3566 | Wild boar (Korean) | Korea | M | SRS703307 | SRR1581058, SRR1581057 |
| WBKR3567 | Wild boar (Korean) | Korea | M | SRS703308 | SRR1581061, SRR1581060 |
| WBKR3568 | Wild boar (Korean) | Korea | M | SRS703309 | SRR1581064, SRR1581063 |
| WBKR3569 | Wild boar (Korean) | Korea | F | SRS703310 | SRR1581067, SRR1581066 |
| WBKR3570 | Wild boar (Korean) | Korea | M | SRS703311 | SRR1581070, SRR1581069 |
| WBKR3571 | Wild boar (Korean) | Korea | F | SRS703312 | SRR1581073, SRR1581072 |
| WBKR3572 | Wild boar (Korean) | Korea | M | SRS703313 | SRR1581075, SRR1581074 |
| WBKR3573 | Wild boar (Korean) | Korea | M | SRS703314 | SRR1581078, SRR1581077 |
| WBKR3574 | Wild boar (Korean) | Korea | F | SRS703315 | SRR1581080, SRR1581081 |
| WBKR3575 | Wild boar (Korean) | Korea | M | SRS703316 | SRR1581084, SRR1581085 |
| K8-11 | KNP | Korea | NA | K8-11 | K8-11_original (not registered) |
| K8-39 | KNP | Korea | NA | K8-39 | K8-39_original (not registered) |
| J-3016 | KNP | Korea | NA | J-3016 | J-3016_original (not registered) |
| J-16 | KNP | Korea | NA | J-16 | J-16_original (not registered) |
| J-17 | KNP | Korea | NA | J-17 | J-17_original (not registered) |
| K-06 | KNP | Korea | NA | K-06 | K-06_original (not registered) |
| K-23 | KNP | Korea | NA | K-23 | K-23_original (not registered) |
| K5-7 | KNP | Korea | NA | K5-7 | K5-7_original (not registered) |
| K5-10 | KNP | Korea | NA | K5-10 | K5-10_original (not registered) |
| K5-22 | KNP | Korea | NA | K5-22 | K5-22_original (not registered) |
| MS20U10 | Meishan | China | F | ERS177331 | ERR173199 |
| MS20U11 | Meishan | China | F | ERS177332 | ERR173200 |
| MS21M07 | Meishan | China | M | ERS177333 | ERR173201 |
| MS21M14 | Meishan | China | M | ERS177334 | ERR173202 |
| MS21M05 | Meishan | China | NA | ERS804955 | ERR977125 |
| MS21M08 | Meishan | China | NA | ERS804957 | ERR977130 |
| MSCN20M03 | Meishan | China | NA | ERS804949 | ERR977109 |
| MSCN20M05 | Meishan | China | NA | ERS804950 | ERR977112 |
| MSCN20U13 | Meishan | China | NA | ERS804953 | ERR977119 |
| MSCN21M01 | Meishan | China | NA | ERS804954 | ERR977122 |
| TCCN3149 | Toncheng | China | F | SRS559502 | SRR1216636 |
| TCCN3148 | Toncheng | China | F | SRS559501 | SRR1216635 |
| TCCN3147 | Toncheng | China | F | SRS559500 | SRR1172577 |
| TCCN3146 | Toncheng | China | F | SRS559499 | SRR1172563 |
| WBCH26M09 | Wild boar (European) | Switzerland | M | ERS177350 | ERR173218 |
| WBES0717 | Wild boar (European) | Spain | M | SRS655622 | SRR1513306 |
| WBES0494 | Wild boar (European) | Spain | M | SRS1541812 | SRR3745077 |
| WBFR25U11 | Wild boar (European) | France | M | ERS177349 | ERR173217 |
| WBIT44U06 | Wild boar (European) | Italy |  | ERS805036 | ERR977380 |
| WBIT44U07 | Wild boar (European) | Italy |  | ERS805037 | ERR977383 |
| WBIT28M39 | Wild boar (European) | Italy | M | ERS805028 | ERR977356 |
| WBNL21F05 | Wild boar (European) | Netherlands | M | ERS177345 | ERR173213 |
| WBNL21M03 | Wild boar (European) | Netherlands | M | ERS177346 | ERR173214 |
| WBTN0965 | Wild boar (European) | Tunis | F | SRS1541813 | SRR3745078 |
| IBGU1805 | Iberian (European domestic) | Spain | M | SRS2170012 | SRR5515065 |
| IBGM0327 | Iberian (European domestic) | Spain | M | SRS655955 | SRR1513307 |
| IBGU1330 | Iberian (European domestic) | Spain | F | SRX245748 | IBGU1330 |
| IBGU1803 | Iberian (European domestic) | Spain | M | SRS1541814 | SRR3745079 |
| IBGU1802 | Iberian (European domestic) | Spain | M | SRX245748 | IBGU1802 |
| IBGU1804 | Iberian (European domestic) | Spain | M | SRS875335 | SRR1917381 |
| LW22F02 | Large White (European domestic) | NA | F | ERS177318 | ERR173186 |
| LW22F03 | Large White (European domestic) | NA | F | ERS177319 | ERR173187 |
| LW22F04 | Large White (European domestic) | NA | F | ERS177320 | ERR173188 |
| LW22F06 | Large White (European domestic) | NA | F | ERS177321 | ERR173189 |
| LW22F07 | Large White (European domestic) | NA | F | ERS177322 | ERR173190 |
| LW22M04 | Large White (European domestic) | NA | M | ERS177323 | ERR173191 |
| LW22M07 | Large White (European domestic) | NA | M | ERS177324 | ERR173192 |
| LW36F01 | Large White (European domestic) | NA | F | ERS177325 | ERR173193 |
| LW36F02 | Large White (European domestic) | NA | F | ERS177326 | ERR173194 |
| LW36F03 | Large White (European domestic) | NA | F | ERS177327 | ERR173195 |
| L5-09 | Landrace (European domestic) | Korea | NA | L5-09 | L5-09_original (not registered) |
| L5-21 | Landrace (European domestic) | Korea | NA | L5-21 | L5-21_original (not registered) |
| L5-27 | Landrace (European domestic) | Korea | NA | L5-27 | L5-27_original (not registered) |
| L5-29 | Landrace (European domestic) | Korea | NA | L5-29 | L5-29_original (not registered) |
| L5-34 | Landrace (European domestic) | Korea | NA | L5-34 | L5-34_original (not registered) |
| L5-40 | Landrace (European domestic) | Korea | NA | L5-40 | L5-40_original (not registered) |
| L5-49 | Landrace (European domestic) | Korea | NA | L5-49 | L5-49_original (not registered) |
| L5-68 | Landrace (European domestic) | Korea | NA | L5-68 | L5-68_original (not registered) |
| L5-84 | Landrace (European domestic) | Korea | NA | L5-84 | L5-84_original (not registered) |
| L5-89 | Landrace (European domestic) | Korea | NA | L5-89 | L5-89_original (not registered) |
| D10-27 | Duroc (European domestic) | Korea | NA | D10-27 | D10-27_original (not registered) |
| D10-28 | Duroc (European domestic) | Korea | NA | D10-28 | D10-28_original (not registered) |
| D10-31 | Duroc (European domestic) | Korea | NA | D10-31 | D10-31_original (not registered) |
| D10-33 | Duroc (European domestic) | Korea | NA | D10-33 | D10-33_original (not registered) |
| D10-34 | Duroc (European domestic) | Korea | NA | D10-34 | D10-34_original (not registered) |
| D10-36 | Duroc (European domestic) | Korea | NA | D10-36 | D10-36_original (not registered) |
| D10-39 | Duroc (European domestic) | Korea | NA | D10-39 | D10-39_original (not registered) |
| D10-41 | Duroc (European domestic) | Korea | NA | D10-41 | D10-41_original (not registered) |
| D10-42 | Duroc (European domestic) | Korea | NA | D10-42 | D10-42_original (not registered) |
| D10-45 | Duroc (European domestic) | Korea | NA | D10-45 | D10-45_original (not registered) |
| BKGB1110 | Birkshire (European domestic) | United Kingdom | F | SRS488257 | SRR1004277 |
| BKGB1111 | Birkshire (European domestic) | United Kingdom | F | SRS488258 | SRR1004278 |
| BKGB1112 | Birkshire (European domestic) | United Kingdom | F | SRS488259 | SRR1004279 |
| BKGB01F10 | Birkshire (European domestic) | United Kingdom | NA | ERS804976 | ERR977197 |
| BKGB01M20 | Birkshire (European domestic) | United Kingdom | NA | ERS804977 | ERR977199 |
